# Exciting Complexity: The Role of Motor Circuit Elements in ALS Pathophysiology

**DOI:** 10.3389/fnins.2020.00573

**Published:** 2020-06-17

**Authors:** Zeynep I. Gunes, Vanessa W. Y. Kan, XiaoQian Ye, Sabine Liebscher

**Affiliations:** ^1^Institute of Clinical Neuroimmunology, Klinikum der Universität München, Ludwig Maximilians University Munich, Munich, Germany; ^2^Graduate School of Systemic Neurosciences, Ludwig Maximilians University Munich, Munich, Germany; ^3^Biomedical Center, Ludwig Maximilians University Munich, Munich, Germany; ^4^Munich Cluster for Systems Neurology (SyNergy), Munich, Germany

**Keywords:** Amyotrophic lateral sclerosis, excitability, upper motor neurons, lower motor neurons, interneurons, astrocytes, neural circuits, excitotoxicity

## Abstract

Amyotrophic lateral sclerosis (ALS) is a fatal disease, characterized by the degeneration of both upper and lower motor neurons. Despite decades of research, we still to date lack a cure or disease modifying treatment, emphasizing the need for a much-improved insight into disease mechanisms and cell type vulnerability. Altered neuronal excitability is a common phenomenon reported in ALS patients, as well as in animal models of the disease, but the cellular and circuit processes involved, as well as the causal relevance of those observations to molecular alterations and final cell death, remain poorly understood. Here, we review evidence from clinical studies, cell type-specific electrophysiology, genetic manipulations and molecular characterizations in animal models and culture experiments, which argue for a causal involvement of complex alterations of structure, function and connectivity of different neuronal subtypes within the cortical and spinal cord motor circuitries. We also summarize the current knowledge regarding the detrimental role of astrocytes and reassess the frequently proposed hypothesis of glutamate-mediated excitotoxicity with respect to changes in neuronal excitability. Together, these findings suggest multifaceted cell type-, brain area- and disease stage- specific disturbances of the excitation/inhibition balance as a cardinal aspect of ALS pathophysiology.

## Introduction

Amyotrophic lateral sclerosis (ALS) is a devastating neurodegenerative disease primarily characterized by the death of upper motor neurons (UMN) and lower motor neurons (LMN) (Kiernan et al., 2011). Upon diagnosis patients only live up to 3−5 years, suffering from progressive paralysis and eventually die from respiratory failure (Cervenakova et al., 2000; Taylor et al., 2016). ALS is the most common form of adult motor neuron diseases with an incidence of ∼2/100,000 per year (Marin et al., 2017) and a prevalence of 2−5/100,000 people (Chiò et al., 2013). The majority of ALS cases (90−95%) occur sporadically (sALS) with unknown etiology, while a mere 5−10% are classified as familial (fALS), out of which only about 40−60% are related to already known mutations (Chen S. et al., 2013; Mejzini et al., 2019). To date, more than 50 genes and gene variants have already been identified in ALS patients (Taylor et al., 2016; Mejzini et al., 2019). The most common forms of fALS are caused by either a hexanucleotide repeat expansion in the chromosome 9 open reading frame 72 (*C9orf72*, ∼40% of all fALS cases), mutations in the superoxide dismutase 1 (*SOD1*, ∼20%), in the TAR DNA-binding protein 43 (*TARDBP*, ∼4%) or in the fused in sarcoma (*FUS*, ∼3%) gene (Renton et al., 2014; Mejzini et al., 2019). One key pathological hallmark of ALS is intracellular protein aggregation, largely as a result of protein misfolding and/or cytosolic mislocalization (Blokhuis et al., 2013; Tyzack et al., 2019). Mechanistically, a number of molecular processes has been proposed to be causally related protein aggregation and motor neuron (MN) death, such as impaired RNA processing (Polymenidou et al., 2012) and proteostasis (Ruegsegger and Saxena, 2016), intracellular Ca^2+^ dyshomeostasis and reduced Ca^2+^ buffering capacity (Grosskreutz et al., 2010; Kawamata and Manfredi, 2010; Leal and Gomes, 2015), cytoskeletal derangements/axonal transport deficits (Xiao et al., 2006; Marinković et al., 2012), mitochondrial dysfunction (Kawamata and Manfredi, 2010), oxidative stress (Barber and Shaw, 2010), and excitotoxicity (Van Den Bosch et al., 2006) to name a few. In addition to those cell autonomous processes, i.e., processes that occur within the affected population of MN, there is also ample evidence pointing toward non-cell autonomous processes, conferred e.g., by glia cells, which strongly regulate disease onset and progression (Boillée et al., 2006; Philips and Rothstein, 2014; Serio and Patani, 2018). These research findings have spurred the development of numerous potential therapeutic substances with anti-glutamatergic, anti-inflammatory, anti-oxidative or neuroprotective effects, which have been tested for the treatment of ALS (Petrov et al., 2017). Amongst these compounds, only Riluzole (anti-glutamatergic) (Bensimon et al., 1994) and recently Edaravone (anti-oxidative) were approved by the FDA (Abe et al., 2014). However, these two compounds offer only very moderate benefits (Petrov et al., 2017; Jaiswal, 2019). A much-improved mechanistic insight into disease pathophysiology is thus urgently needed in order to identify novel, effective therapeutic approaches to combat ALS. One intriguing and consistent finding in both mouse models of the disease (Kim et al., 2017; Martínez-Silva et al., 2018) as well as in human patients (Kanai et al., 2006; Tamura et al., 2006; Vucic et al., 2008; Menon et al., 2015, 2017; Cengiz et al., 2019) are changes in neuronal excitability, which have been proposed to represent one of the earliest modifications in a cascade of pathological events leading to eventual MN death. The mechanisms underlying these excitability changes, as well as their downstream consequences are still incompletely understood, but hold the great promise for an early diagnosis and the identification of novel treatment options. In this review, we will thus summarize the current knowledge regarding alterations of excitability and activity of affected upper and lower motor neurons in humans and rodent models of the disease. Next, we will recapitulate molecular, structural and functional changes found in direct or indirect neuronal input partners of affected MNs and we will address alterations found in astrocytes, which also play an important role in the regulation of MN excitability and health.

## What is “*Excitability*” and How is It Assessed in Humans and Rodent Models of ALS?

The term *excitability* refers to a neuron’s propensity to generate an output [change in membrane potential, typically in the form of an action potential (AP), Box 1] in response to an input exceeding a certain threshold. This intrinsic property of a neuron (“intrinsic excitability”) is determined by a number of factors that define biophysical properties of the cell, such as the composition, affinity and quantity of receptors (Hou and Zhang, 2017; Terunuma, 2018), pores or channels (e.g., K^+^ and Na^+^ channels) (Rutecki, 1992; Edwards and Weston, 1995; Schulz et al., 2006; Lin and Baines, 2015). When investigating the excitability or activity of individual neurons or neuronal populations, different methodological approaches and read-outs can be employed. The excitability of individual neurons can be assessed e.g., by intracellular recordings or patch-clamping (Cowan and Wilson, 1994; Hutcheon et al., 1996; Uusisaari et al., 2007; Gentet et al., 2010; Segev et al., 2016) (see Box 2). The excitability of multiple neurons or neuronal populations, on the other hand, can be monitored by extracellular field recordings (Jun et al., 2017; De Franceschi and Solomon, 2018) or optical means, such as voltage sensitive dyes (Kuhn et al., 2008; Akemann et al., 2013) or calcium indicators (Stosiek et al., 2003; Chen J.L. et al., 2013). To probe the excitability of neurons in humans, more indirect measures are typically employed, such as a combination of transcranial magnetic stimulation (TMS) together with electroencephalography (EEG) (Miniussi et al., 2012; Hill et al., 2016; Gonzalez-Escamilla et al., 2018) or for the motor system TMS stimulation of the motor cortex and simultaneous recording of the motor-evoked potential (MEP) of the respective innervated muscle. The actual activity of neurons (that is the frequency of APs) hinges on a number of factors, including the intrinsic excitability of a neuron, strength of individual synapses, as well as on the quantity and timing of excitatory synaptic input and its regulation by inhibition, a phenomenon called excitation-inhibition (E/I) balance (He and Cline, 2019; Kiernan et al., 2019). It is important to note that altered excitability is not necessarily reflected in altered neuronal activity. Alterations in intrinsic excitability can be compensatory in response to reduced input to re-establish former activity levels (e.g., after sensory deprivation) (Hengen et al., 2013; Lambo and Turrigiano, 2013). Under pathological conditions, changes in excitability can be concordant or discordant with changes in neuronal activity (Le Feber et al., 2014; Busche and Konnerth, 2015; Kim et al., 2017). To get to the heart of the matter, it is thus important to assess not only intrinsic properties of a neuron, but to also characterize and quantify synaptic inputs and measure effective activity levels – ideally *in vivo* in the intact CNS.

**Box 1. Definitions of neuronal hyperexcitability and hyperactivity.**

***Hyperexcitability***: Excitability is an electrophysiological property of a neuron, referring to its propensity to depolarize its membrane potential upon a given stimulus. It can be assessed e.g., by measuring the current needed to cause an AP (rheobase) or the frequency of fired APs in response to a defined input (frequency-current curve, F-I curve). Hyperexcitability is used to characterize a neuron, which is capable of producing a significantly larger AP frequency upon a defined input/stimulus compared to the majority of neurons of the same type under control conditions.

***Hyperactivity***: Neuronal activity refers to the frequency of fired AP (typically spontaneously elicited APs − that is without a defined stimulation or in case of awake mice in the absence of any behavior), which can be measured either by electrophysiological recordings or optical means employing Ca^2+^ indicators to probe changes in intracellular calcium in the form of fluorescent signals (transients) as a proxy for neuronal activity. A cell is considered hyperactive if it fires more APs (or Ca^2+^ transients) than the majority of neurons of the same type under identical conditions. The effective activity cut off defining hyperactivity is so far applied heuristically (Busche et al., 2015, 2008; Lerdkrai et al., 2018; Burgold et al., 2019).

**Box 2. Techniques and terminologies used in clinical studies measuring cortical excitability in ALS patients.**

***Transcranial magnetic stimulation* (*TMS*)**: A non-invasive procedure of applying a local time-varying magnetic field using a stimulation coil to depolarize neurons beyond their AP firing threshold. For the assessment of motor cortex excitability it is coupled with the measurement of motor evoked potentials (MEP), recorded from a contralateral innervated muscle (e.g., abductor pollicis brevis muscle) (Vucic et al., 2013).

***Threshold-tracking TMS***: The electromagnetic stimulus intensity required to maintain a target MEP response of 200 μV (motor threshold) is measured, reflecting the excitability of UMN (Fisher et al., 2002; Vucic et al., 2018; Cengiz and Kuruoğlu, 2020).

***Resting motor threshold* (*RMT*)**: The lowest electromagnetic stimulus intensity required to produce an MEP of at least 50 μV at rest (Rothwell et al., 1999; Rosso and Lamy, 2018).

***Intracortical facilitation* (*ICF*)**: Increased excitability primed by a conditioning stimulus. The MEP is measured upon the application of a paired pulse electromagnetic stimulation at an interstimulus interval of 7−30 ms (Wagle-Shukla et al., 2009).

***Short-interval intracortical inhibition* (*SICI*)**: Inhibition of the MEP response upon a conditioning stimulus applied at a short latency (7−10 ms) prior to the actual stimulation. It compares the MEP amplitude with and without a preceding subthreshold conditioning stimulus (Wagle-Shukla et al., 2009; Cengiz and Kuruoğlu, 2020).

***Cortical silent-period* (*CSP*) *duration***: Assessment of the duration a voluntary muscle contraction (as measured be EMG) is interrupted by a previous TMS stimulation (typically 100−300 ms upon TMS stimulation). It is considered a functional assessment of intracortical inhibition, elicited by the activation of GABAergic interneurons (Cantello et al., 1992; Vucic et al., 2008; Poston et al., 2012).

### Excitability Changes of UMN

Research of the past decade has identified altered excitability of UMN and LMN in ALS, both in human patients as well as in rodent models of the disease or cell culture systems. Notably, it has been suggested that the pathology is initiated in the motor cortex and propagates further to the spinal cord, forming the basis of the “dying forward hypothesis” (Eisen and Weber, 2001; Braak et al., 2013). Alternatively, the “dying-backward hypothesis,” proposed by others, posits that the disease is initiated in the muscle or the neuromuscular junction (NMJ), from where it retrogradely affects LMNs and subsequently UMNs in cortex (Kiernan et al., 2011; Baker, 2014). Whether or not there is one common mode of disease-initiation shared by all forms of ALS, remains unanswered to date. Nonetheless, changes in excitability have been reported for both systems and shall be summarized here.

**FIGURE 1:**
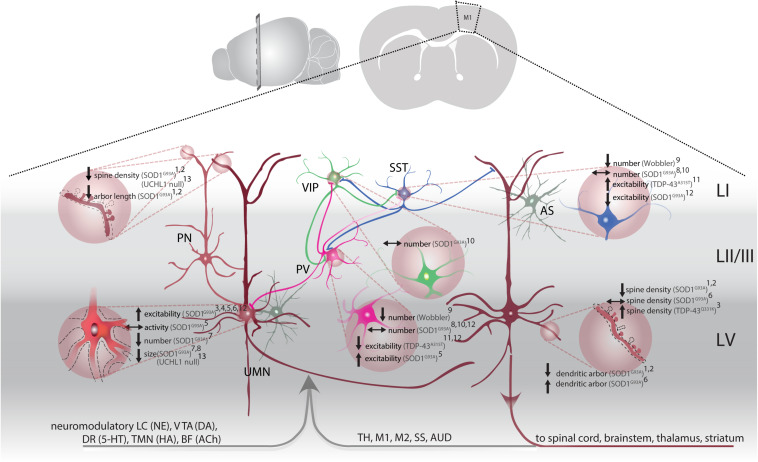
M1 circuitry and pathophysiological changes in ALS. Local (glutamatergic, excitatory) input to upper motor neurons (UMN, brown) is mainly provided by upstream LII/III pyramidal neurons (PN, light brown) and modulated by astrocytes (AS, gray). Parvalbumin (PV, magenta), somatostatin (SST, blue) and vasoactive intestinal peptide (VIP, green) interneurons provide GABAergic input within M1. PV and SST target PN, including UMNs, as well as inhibit each other (inhibition of PV by SST more frequent). VIP are disinhibitory by synapsing on PV and SST. Long-range input to M1 originates from cortical and subcortical structures: thalamus (TH), primary motor cortex (M1), secondary motor cortex (M2), somatosensory cortex (SS), auditory cortex (AUD). Neuromodulatory input stems from: the locus coeruleus [LC, releasing norepinephrine (NE)], ventral tegmental area [VTA, releasing dopamine (DA)], dorsal raphe [DR, releasing serotonin (5-HT)], tuberomammillary nucleus [TMN, releasing histamine (HA)] and basal forebrain [BF, acetylcholine (ACh)]. UMN project to the spinal cord, brainstem and send axon collaterals to the thalamus and striatum. Pathological changes (light brown filled circles) are identified throughout the M1 microcircuitry: Structural changes, e.g., altered spine density and dendritic regression are observed on apical dendrites of LII/III PN^1,2^ and on apical and basal dendrites of UMN^1,2^, along with a reduction in overall number^7^ and soma size^7,8,12^. UMN are hyperexcitable^3,4,5,6^, but don’t display overall activity changes^5^. Interneuron density was affected: while PV and SST are reduced in Wobbler mice^9^, density of all three interneuron subtypes remained unchanged in SOD1^G93A^ mice^8,10^. Excitability of PV and SST was altered differentially. While hyperexcitability was observed in PV of SOD1^G93A^ mice^5^, hypoexcitable PV were accompanied by hyperexcitable SST in TDP-43^A315T^ mice^11^. ^1^(Fogarty et al., 2016b); ^2^(Fogarty et al., 2015); ^3^(Fogarty et al., 2016a); ^4^(Pieri et al., 2009); ^5^(Kim et al., 2017); ^6^(Saba et al., 2016); ^7^(Zang and Cheema, 2002); ^8^(Özdinler et al., 2011); ^9^(Nieto-Gonzalez et al., 2011); ^10^(Clark et al., 2017); ^11^(Zhang et al., 2016); ^12^(Gautam et al., 2016).

What is the evidence for excitability changes of UMN? In ALS patients, there is compelling evidence that motor cortex (M1) is hyperexcitable (Ziemann et al., 1997; Zanette et al., 2002; Vucic et al., 2008, 2009; Menon et al., 2015, 2017; Shibuya et al., 2017; Van den Bos et al., 2018; Cengiz et al., 2019; Table 1). A common method to assess cortical excitability in humans is threshold-tracking transcranial magnetic stimulation (TMS, see Box 2). The approach is based on the application of a local time- varying magnetic field of increasing intensities to gauge the intensity needed to depolarize neurons beyond their firing threshold (Barker et al., 1985; Hess and Ludin, 1988; Hallett, 1996; Oliveri et al., 2000, Oliviero et al., 2011). Although the mechanisms underlying the TMS-triggered depolarization of pyramidal neurons (PN) are still incompletely understood, it is a widely used approach to investigate the excitability of neuronal populations (Kim et al., 2005), such as UMN in humans (Ziemann et al., 1997; Siciliano et al., 1999; Zanette et al., 2002; Turner et al., 2005b; Vucic et al., 2008; Vucic and Kiernan, 2009; Menon et al., 2015, 2017; Shibuya et al., 2017; Van den Bos et al., 2018; Cengiz et al., 2019; Cengiz and Kuruoğlu, 2020). To verify the activation of UMN [or corticospinal excitability; (Cortes et al., 2012)], the motor evoked potential (MEP) of the innervated muscle is recorded (e.g., the abductor pollicis brevis muscle) (Vucic and Kiernan, 2006; Van den Bos et al., 2018). Alterations in cortical excitability could either be caused by enhanced intrinsic excitability and/or excitation or decreased inhibition. To differentiate those two options, different TMS stimulation protocols were developed to selectively investigate excitatory and inhibitory circuit function. A phenomenon reflecting inhibitory network function is short interval intracortical inhibition (SICI), which is probed by pairing a subthreshold TMS stimulus with a suprathreshold stimulus within a time window of 7−10 ms. The suprathreshold stimulus, needed to evoke a defined MEP, is much higher compared to a stimulation without a preconditioning stimulus (Vucic and Kiernan, 2006; Vucic et al., 2008; Wagle-Shukla et al., 2009; Shirota et al., 2010). Excitatory network function, on the other hand, is tested by assessing intracortical facilitation (ICF). In this case a subthreshold conditioning stimulus is followed by a test stimulus within 10−30 ms (Oliveri et al., 2000; Vucic and Kiernan, 2006). The intensity of the test stimulus, necessary to evoke a defined MEP response, is lower compared to an unconditioned stimulus (Ziemann et al., 1998; Vucic et al., 2008; Box 2). Employing variations of those TMS stimulation protocols studies have identified a reduction in the threshold needed to generate a MEP (Zanette et al., 2002; Menon et al., 2015, 2017), as well as a reduction in the intracortical inhibition (Ziemann et al., 1997; Zanette et al., 2002; Turner et al., 2005a, b; Vucic et al., 2008, 2009; Vucic and Kiernan, 2009; Menon et al., 2015, 2017; Shibuya et al., 2017; Van den Bos et al., 2018; Cengiz et al., 2019; Cengiz and Kuruoğlu, 2020) and an increase in the intracortical facilitation (Zanette et al., 2002; Vucic et al., 2008; Vucic and Kiernan, 2009; Menon et al., 2017; Van den Bos et al., 2018), thereby establishing that M1 E/I imbalance in ALS is based on a combination of increased excitability and decreased inhibition (Table 1). Remarkably, there is evidence that cortical hyperexcitability precedes the actual onset of UMN and LMN symptoms, thus arguing in favor of a cortical origin of ALS (Vucic et al., 2008). What is known about the underlying molecular mechanisms causing cortical hyperexcitability? Studies employing proton magnetic resonance spectroscopy, positron emission tomography (PET) imaging or postmortem immunohistochemistry and whole-genome sequencing unraveled increased tissue levels of glutamate-glutamine and reduced levels of GABA (Foerster et al., 2012; Foerster et al., 2013; Khademullah et al., 2020), lower PV-expressing interneuron count (Khademullah et al., 2020), decreased GABA_A_ receptor densities, changes in GABA_A_ receptor composition (reduced α1 subunit and increased β1 subunit expression) (Petri et al., 2003), downregulation of NMDA receptor subunits and dysregulation of AMPA receptors in M1 (Aronica et al., 2015). What are the cellular or circuit mechanisms underlying the observed changes in cortical excitability? Transgenic (tg) mouse models of ALS are a valuable tool to address this question (Table 2). The most frequently used model in ALS research is the SOD1^G93A^ tg mouse (Gurney et al., 1994), which has been extensively characterized in the past decades. However, studying UMN pathology in rodent models is hampered by the fact that the motor system is wired up differently, such that UMN do not monosynaptically impinge on LMN (Anderson et al., 2010; Kaneko, 2013; Shepherd, 2013) and mouse models of the disease only partly mimic the degeneration of UMN. Thus, for quite some time it had been questioned as to whether UMN degeneration is recapitulated rodent models of the disease at all. In order to demonstrate UMN involvement in rodent models, Zang and Cheema (2002) and Özdinler et al. (2011) characterized the abundance and signs of UMN degeneration in the SOD1^G93A^ mouse model. They found a reduction of UMN quantity (identified upon retrograde labeling) early during the presymptomatic stage, coinciding with evidence for UMN apoptosis and UMN somata size reduction, even a month prior to changes in overall numbers (Figure 1). Furthermore, layer V PN in the SOD1^G93A^ mouse model are also affected structurally (Fogarty et al., 2015), as seen in a regression of apical dendrites and a reduction in the density of dendritic spines, the structural correlates of post-synapses, on apical and basal dendrites of layer V neurons (Fogarty et al., 2015, 2016b; Figure 1). Importantly, these abnormalities occurred early presymptomatically (P21−P30) and persisted until late in life in this mouse model (Fogarty et al., 2015, 2016b). Similar findings were observed in another model, namely UCHL1 -/- mice, which carry an intragenic deletion within the ubiquitin carboxy-terminal hydrolase L1 gene. These mice display clear signs of UMN degeneration, including vacuolated apical dendrites, dendritic regression and spine loss (Jara et al., 2015). Contrary to those reports TDP-43^Q331K^ tg mice, expressing TDP-43 with a Q331K mutation, seem to possess even a greater spine density of layer V PN than the WT controls already early presymptomatically (P30) (Fogarty et al., 2016a; Figure 1). The investigation of other, novel or less frequently studied, mouse models of ALS has yielded mixed results. While most of them present with motor symptoms, coinciding with neuronal degeneration in the spinal cord, assessment of cortical pathology has either not been conducted in detail yet or yielded variable results. As such, Alsin^KO^ (Gautam et al., 2016), hPFN1 ^G118V^ (Fil et al., 2017), Prp-TDP43^A315T^ (Wegorzewska et al., 2009) or transgenic models of C9orf72 hexanucleotide repeat expansion (Batra and Lee, 2017) show signs of UMN degeneration, but a detailed morphological investigation has not been performed yet (see Supplementary Table 1 for overview of mouse models).

**TABLE 1:** Summary of excitability changes reported in motor cortex of ALS patients.

**ALS type**	**Age (average)**	**Method**	**Finding**	**References**
sALS	63.8 years	paired-pulse TMS	hyperexcitability & inhibition **↓**: MEP_max_ **↓**, ICI **↓**	(Ziemann et al., 1997)
	65.3 years	threshold-tracking TMS	compromised inhibition: MEP onset latency **↑**, CSP duration **↑**/**↓**, early stages: CSP duration **↑**, later stages: CSP duration **↓**	(Siciliano et al., 1999)
	61.4 years	single pulse and paired-pulse threshold tracking TMS	inhibition **↓**: MEP amplitude **↔**, RMT **↔**, ICF **↔**, ICI **↓**, CSP duration **↓**	(Zanette et al., 2002)
	56.3 years	microarray, RT-qPCR of post-mortem tissue	various transcriptional alterations, including NMDA and AMPA receptors	(Aronica et al., 2015)
	62.5 years	threshold-tracking TMS	hyperexcitability in cortex (effect more prominent contralateral to site of disease onset) & inhibition **↓**: MEP amplitude **↑**, RMT **↓**, SICI **↓**, CSP duration **↓**	(Menon et al., 2017)
sALS and fALS (hom SOD1^D90A^)	57 years (sALS), 53 years (fALS)	single pulse and paired-pulse threshold tracking TMS; [^11^C]flumazenil PET (GABA_A_ receptor ligand)	inhibition **↓**: SICI **↓**, [^11^C]flumazenil binding **↓**, neuronal loss/dysfunction **↑**	(Turner et al., 2005a, b)
fALS (SOD1, asymp. and symp.)	40 years (asymp.), 58.7 years (symp.)	threshold-tracking TMS	hyperexcitability and inhibition **↓**: MEP amplitude **↑**, ICF **↑**, SICI **↓**, stimulus-response curve **↑**	(Vucic et al., 2008)
n.s.	67 years	ISH histochemistry on human postmortem M1	inhibition **↓**: GABA_A_ α1 subunit mRNA **↓**, GABA_A_ β1-subunit mRNA **↑**	(Petri et al., 2003)
	59.5 years	3-T proton magnetic resonance spectroscopy	excitatory & inhibitory NT imbalance: GABA **↓**, Glu **↓** (in Riluzole-treated group)	(Foerster et al., 2013)
	60.2 years	single pulse and paired-pulse threshold tracking TMS	hyperexcitability & inhibition **↓**: MEP amplitude **↑**, RMT**↓**, ICF **↑**, SICI **↓**, CSP duration **↓**	(Zanette et al., 2002; Menon et al., 2015)
	63 years	single pulse and paired-pulse threshold tracking TMS	hyperexcitability & inhibition **↓**: MEP amplitude **↑**, RMT **↔**, SICF **↑**, SICI **↓**, CSP duration **↓**	(Van den Bos et al., 2018)
	59.8 years	threshold-tracking TMS	hyperexcitability & inhibition **↓**: mean SAI and LAI values **↓**, SICI **↓**, MEP/CMAP amplitude ratio **↑**	(Cengiz et al., 2019)
	61.3 years			(Shibuya et al., 2017)
	57.1 years	threshold-tracking TMS	inhibition **↓**: RMT **↔**, ICF **↔**, SICF **↓** (for ISIs 1−1.8 ms & 2−3 ms), SICF **↔** (for ISI 4−4.6 ms), SICI **↓**	(Cengiz and Kuruoğlu, 2020)
fALS	58 years	threshold-tracking TMS	hyperexcitability & inhibition **↓**: SICI **↓**, ICF **↑**	(Vucic and Kiernan, 2009)

*AMPA, a-amino-3-hydroxy-5-methyl-4-isoxazolepropionic acid; asymp., asymptomatic; CMAP, compound muscle action potential; CSP, cortical-silent period; fALS, familial ALS; GABA, γ-aminobutyric acid; GABA_*A*_, γ-aminobutyric acid receptor subtype A; Glu, glutamate; hom, homozygous; SOD1, superoxide dismutase 1; ICF, intracortical facilitation; ISH, *in situ* hybridization; ISI, interstimulus interval; LAI, long latency afferent inhibition; M1, primary motor cortex; MEP, motor evoked potential; MEP_*max*_, maximum motor evoked potential; mRNA, messenger RNA; n.s., not specified; NMDA, N-methyl-D-aspartate; NT, neurotransmitter; PET, positron emission tomography; RT-qPCR, Real-Time quantitative polymerase chain reaction; RMT, resting motor threshold; SAI, short latency afferent inhibition; sALS, sporadic ALS; SICF, short interval intracortical facilitation; SICI, short interval intracortical inhibition; symp., symptomatic; TMS, transcranial magnetic stimulation.*

**TABLE 2:** Excitability related alterations of upper motor neurons (UMN) in rodent ALS models.

**Disease stage**	**ALS model**	**Age**	**Method of investigation**	**Finding**	**References**
presymptomatic	SOD1^G93A^	11−12 DIV	whole-cell patch clamp recordings in neonatal mouse-derived cortical culture	RMP **↔**, input resistance **↔**, hyperexcitability: spiking frequency **↑**, persistent Na^+^ current **↑**	(Pieri et al., 2009)
		P5−P6	whole-cell patch clamp recordings in neonatal brain slice	RMP **↔**, input resistance **↔**, hyperexcitability: rheobase **↓**, max. AP frequency **↑**	(Kim et al., 2017)
		P26−P40	whole-cell patch clamp recordings in brain slice	RMP for CSN **↓**, RMP for CCN**↔**, input resistance, **↔**, spiking frequency **↔**, rheobase **↔**, max. AP frequency **↔**	
		P21−P40	whole-cell patch clamp recordings in brain slice and dye filling	EPSC **↑**, IPSC **↔**, dendritic arbor length **↓**, apical & basal dendritic spine density **↓**	(Fogarty et al., 2015, 2016b)
		P26−P31	whole-cell patch clamp recordings in brain slice, RT-qPCR, WB and IHC	RMP **↔**, input resistance **↔**, hyperexcitability: F-I gain **↑**, rheobase **↓**, sEPSC & mEPSC **↑**, basal dendritic arborization **↑**, basal dendritic spine density **↔**, VGLUT2 mRNA & protein expression **↑**	(Saba et al., 2016)
		P28−P35	Golgi-Cox staining	cortical thickness **↓**, soma volume **↔**, dendritic arbor length, **↓** apical & basal dendritic spine density **↓**	(Fogarty et al., 2016b)
		P60	*in vivo*^1^H-MRS of brain	Gln **↔**, Glu **↔**, GABA **↓**, Gly **↔**, Glx **↔**, Gln/Glu **↔**	(Lei et al., 2019)
	TDP-43^Q331K^	P26−P35	whole-cell patch clamp recordings in brain slice and morphology assessment	EPSC **↑**, IPSC **↔**, apical & basal dendritic spine densities **↑**	(Fogarty et al., 2016a)
symptomatic	SOD1^G93A^	P6−P75	Golgi-Cox staining	cortical thickness **↓**, soma volume **↔**, dendritic arbor length **↓**, apical & basal dendritic spine density **↓**	(Fogarty et al., 2016b)
		P90−P129	whole-cell patch clamp in brain slice	RMP **↔**, hyperexcitability: rheobase **↓**, max. AP frequency **↑**, input resistance **↑**	(Kim et al., 2017)
		P92−P134	*in vivo* two-photon Ca^2+^ imaging in awake mice	neuronal activity **↔**	(Kim et al., 2017)
		P100	*in vivo*^1^H-MRS of brain	Glu **↓**, GABA, Gly, Glx, Gln and Gln/Glu **↔**	(Lei et al., 2019)
		P115		Glu **↓**, GABA **↓**, Gln/Glu **↑**, Gly, Glx and Gln **↔**	(Lei et al., 2019)
		P120−P122		Glu **↓**, Glx **↓**, Gln **↑**, Gln/Glu **↑**, GABA and Gly **↔**	(Lei et al., 2019)
		P120	Golgi-Cox staining	cortical thickness **↓**, soma volume **↔**, dendritic arbor length **↓**, basal dendritic spine density **↓**	(Fogarty et al., 2016b)
		P120−P165	WB, intracerebral dialysis and HPLC of dialysate	[Glu & Asp] **↑**, extracellular Glu clearance (Glu extraction fraction) **↓**, GLT-1, GLAST and EAAC1 expression **↔**	(Alexander et al., 2000; Deitch et al., 2002)

*AP, action potential; Asp, aspartate; CCN, corticocortical neuron; CSN, corticospinal neuron; DIV, days *in vivo*; EAAC1, excitatory amino acid transporter 3; EPSC, excitatory postsynaptic currents; F-I, frequency-current; GABA, γ-aminobutyric acid; GLAST, glutamate–aspartate transporter; Gln, glutamine; Gln/Glu, ratio of glutamine and glutamate; GLT-1, glutamate transporter 1; Glu, glutamate; Glx, sum of glutamate and glutamine; Gly, glycine; HPLC, high performance liquid chromatography; IHC, immunohistochemistry; IPSC, inhibitory post synaptic currents; mEPSC, miniature excitatory postsynaptic currents; motor neuron; mRNA, messenger ribonucleic acid; RT-qPCR, real-time quantitative polymerase chain reaction; RMP, resting membrane potential; sEPSC, spontaneous excitatory postsynaptic currents; SOD1, superoxide dismutase 1; TARDBP, TAR DNA binding protein; tg, transgenic; VGLUT2, vesicular glutamate transporter 2; WB, western blotting.*

Importantly, do these structural alterations translate into functional deficits? To this end, patch-clamp recordings of layer V PN were performed in acute brain slices of early presymptomatic SOD1^G93A^ and TDP-43^Q331K^ tg mice and indeed, an increase in the frequency of excitatory synaptic currents (Fogarty et al., 2015, 2016a,b; Saba et al., 2016), a decrease in rheobase (Saba et al., 2016), but no change in inhibitory synaptic frequency (Fogarty et al., 2015, 2016a,b) was found (Figure 1). Cortical layer V comprises several different populations of PN, which can be classified based on their projection areas. Kim et al. used retrograde labeling to identify UMN amongst other populations and characterized electrophysiological properties at different disease stages in SOD1^G93A^ tg mice (Kim et al., 2017). These experiments revealed disease stage − specific changes: already in neonatal mice UMN were hyperexcitable, seen in a lower rheobase and increased maximal firing frequency. In presymptomatic mice, these changes were normalized and did not differ from WT UMN anymore. In symptomatic mice a hyperexcitable phenotype was observed again (Kim et al., 2017; Buskila et al., 2019), which, however, did not reflect in an actual change in neuronal activity *in vivo* as measured by means of two-photon calcium imaging (Kim et al., 2017; Figure 1). Moreover, RNA-sequencing analysis in SOD1^G93A^ tg mice revealed differential expression of over 300 genes in UMN early postnatally, such as the downregulation of CACNB4 (voltage-dependent L-type calcium channel subunit beta-4) and GABAR4 [γ-aminobutyric acid (GABA) receptor subunit alpha-4] (Kim et al., 2017). Together, these findings indicate that both intrinsic excitability as well as excitatory synaptic input is increased in UMN in mouse models of the disease and that these changes occur very early during the presymptomatic stage of the disease. Changes in excitability, however, do not seem to alter the activity of UMN *in vivo* (Figure 1).

### Excitability Changes of LMN

**FIGURE 2:**
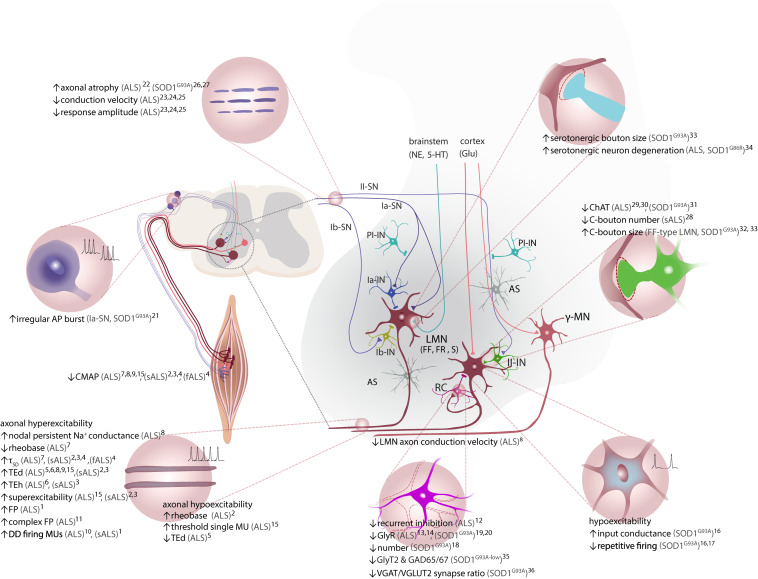
ALS-associated alterations in ventral spinal cord circuitry. LMN receive inhibitory input via Ia-IN, Ib-IN, and RC, and excitatory inputs from cortiospinal tract (UMN), II-IN and SN. γ-motor neurons, which are spared in ALS, do not receive direct inputs from Ia-SN. Excitatory inputs to LMNs via Ia afferent terminals are controlled by PI-IN. Both excitatory and inhibitory inputs are tightly regulated by the proprioceptive feedback provided by sensory afferents (Ia, Ib and II-SN) and astrocytes. Axonal hyperexcitability and hypoexcitability are reported in ALS patients. Decreased RC synapses on LMN and lower number of RC is reported. LMN hypoexcitability is present *in vivo* in SOD1^G93A^ tg mouse model. Ia-SN neurons exhibit irregular firing patterns as an indication of their altered excitability/activity. Cholinergic C-bouton number is decreased in sALS patients, but C-boutons are enlarged especially onto vulnerable FF LMN in SOD1^G93A^ tg mice. Protein and mRNA levels of ChAT are decreased in spinal cord of ALS patients. Reduced ChAT expression is reported in II-IN and C-boutons on MN of SOD1^G93A^ tg mice. Serotonergic boutons on LMN are increased in low-copy SOD1^G93A^ tg mice, whereas serotonergic neurons in brainstem degenerate in both ALS patients and SOD1^G86R^ tg (not shown). Please note that monosynaptic connections between UMN and LMN are only present in humans. Neuromodulatory synapses are depicted as one (somata located in brainstem). CPGs and descending reticulospinal tract projections to LMN via commissural INs are not depicted for simplicity. Studies with unspecified type of ALS are referred to as (ALS). AP, action potential; AS, astrocytes; CMAP, compound muscle action potential; ChAT, choline acetyltransferase; CPG, central pattern generator; DD, double-discharge; fALS, familial ALS; FF, fast-fatigable; FP, fasciculation potential; FR, fast-resistant; gamma (γ)-motor neuron, γ-MN; GAD65/67, glutamic acid decarboxylase 65/67; Glu, glutamate; GlyT2, glycine transporter 2; Ia-/Ib-IN, class Ia/Ib spinal interneuron; II-IN, class II spinal interneuron; LMN, lower motor neuron; MU, motor unit; NE, norepinephrine; Ia-/Ib-SN, class Ia/Ib sensory neuron; II-SN, class II sensory neuron; PI-IN, presynaptic inhibitory interneuron; RC, Renshaw cell; sALS, sporadic ALS; SOD1, superoxide dismutase 1; S, slow; TEd, depolarizing threshold electrotonus; TEh, hyperpolarizing threshold electrotonus; τ_SD_, strength-duration constant, UMN, upper motor neuron; VGAT, vesicular GABA transporter; VGLUT, vesicular glutamate transporter 2; 5-HT, serotonin. ^1^(Kostera-Pruszczyk et al., 2002); ^2^(Kanai et al., 2006); ^3^(Vucic and Kiernan, 2006); ^4^(Vucic et al., 2009); ^5^(Bostock et al., 1995); ^6^(Horn et al., 1996); ^7^(Mogyoros et al., 1998); ^8^(Tamura et al., 2006); ^9^(Nakata et al., 2006); ^10^(Piotrkiewicz et al., 2008); ^11^(de Carvalho and Swash, 2013); ^12^(Raynor and Shefner, 1994); ^13^(Hayashi et al., 1981); ^14^(Whitehouse et al., 1983); ^15^(Howells et al., 2018); ^16^(Delestrée et al., 2014); ^17^(Martínez-Silva et al., 2018); ^18^(Chang and Martin, 2009); ^19^(Chang and Martin, 2011a); ^20^(Chang and Martin, 2011b); ^21^(Seki et al., 2019); ^22^(Heads et al., 1991); ^23^(Hammad et al., 2007); ^24^(Pugdahl et al., 2007); ^25^(Sangari et al., 2016); ^26^(Guo et al., 2009); ^27^(Sassone et al., 2016); ^28^(Nagao et al., 1998); ^29^(Oda et al., 1995); ^30^(Virgo et al., 1992); ^31^(Casas et al., 2013); ^32^(Pullen and Athanasiou, 2009); ^33^(Saxena et al., 2013); ^34^(Dentel et al., 2012); ^35^(Hossaini et al., 2011); ^36^(Sunico et al., 2011).

What is known about the electrophysiological properties of LMN in ALS patients? The investigation of LMN activity/excitability in humans largely relies on indirect measures, such as nerve conduction studies (NCS) and electromyography (EMG) (Joyce and Carter, 2013; Box 3 and Supplementary Table 2). These studies revealed an increase in motor unit excitability, evidenced by the increased presence of fasciculation potentials, double discharges of the motor unit (Kostera-Pruszczyk et al., 2002; Piotrkiewicz et al., 2008) and aberrant single motor unit firing (Piotrkiewicz et al., 2008) and increased axonal excitability (Bostock et al., 1995; Kanai et al., 2006; Nakata et al., 2006; Figure 2). Increased axonal excitability in ALS is likely due to enhanced persistent axonal Na^+^ conductance and impairments in axonal K^+^ conductance (Bostock et al., 1995; Horn et al., 1996; Mogyoros et al., 1998; Kanai et al., 2006; Nakata et al., 2006; Tamura et al., 2006; Vucic and Kiernan, 2006, 2009; Box 3) and was suggested to contribute to fasciculation potentials typical of ALS (de Carvalho and Swash, 2013; Howells et al., 2018; Figure 2 and Supplementary Table 2). However, others showed that fasciculations cannot be solely explained by increased Na^+^ conductance, but must rely on impairments of all ion channels, including reduced inward and outward rectifying K^+^ channels (Howells et al., 2018). Nevertheless, these studies can only provide indirect measures of overall neuronal excitability. Indeed, Nakata et al. reported that distal parts of the axon display more prominent K^+^ channel dysfunction than the nerve trunk, thus hyperexcitability is more evident in nerve terminals (Nakata et al., 2006).

**Box 3. Techniques used to assess LMN excitability in ALS patients and common terminology.**

***Electromyography* (*EMG*)**: Measurement of electrical activity (voltage change) in a muscle. Parameters assessed are the frequency, amplitude and shape of signals, and whether they occur spontaneously. EMG recordings are decisive for the differentiation between neurogenic or myogenic lesions. Signs typical of a neurogenic lesion (as in ALS) are abnormal spontaneous activity, presence of fasciculation potentials and fibrillations, reduced motor unit recruitment and motor unit potentials with greater amplitude or duration to name a few (Joyce and Carter, 2013). These parameters set diagnostic criteria for ALS defined by the revised El Escorial criteria (Ludolph et al., 2015).

***Nerve conduction studies* (*NCS*)**: Synonymous to nerve conduction velocity (NCV). NCS assess the velocity of an applied electrical signal propagating along a peripheral nerve. It is often combined with EMG to also measure the compound muscle action potential (CMAP). While ALS patients do not exhibit demyelination, thus have normal nerve conduction velocity, decreased CMAP is commonly seen (Mogyoros et al., 1998; Kanai et al., 2006; Tamura et al., 2006; Vucic and Kiernan, 2006; Vucic and Kiernan, 2009), which primarily indicates a reduction in intact motor axons innervating the respective muscle (Mallik and Weir, 2005).

**FIGURE 3:**
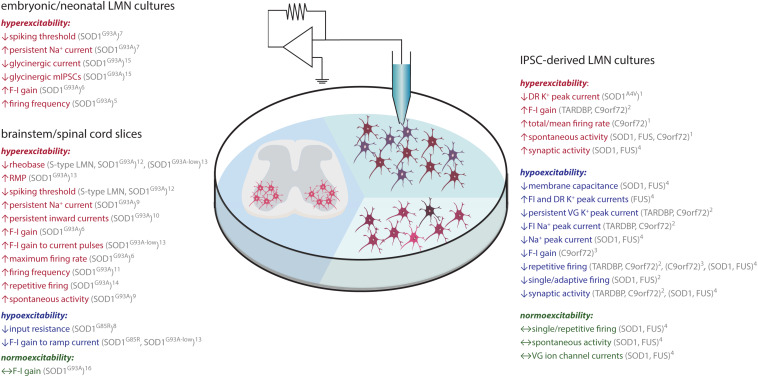
ALS-associated changes in LMN excitability assessed *in vitro*. Findings obtained through recordings of LMN (brown) in embryonic/neonatal cell culture, IPSC-derived LMN culture or spinal cord slice culture are shown. Data indicative of hyperexcitability is shown in red, hypoexcitability in blue and no change in green. Abbreviations: C9orf72, chromosome 9 open reading frame 72; DR, delayed-rectifying; FI, fast -inactivating; F-I, frequency-current; FUS, fused in sarcoma; IPSC, induced pluripotent stem cell; LMN, lower motor neuron; RMP, resting membrane potential; SOD1, superoxide dismutase 1; TDP-43, TAR DNA-binding protein 43; VG, voltage-gated. ^1^(Wainger et al., 2014); ^2^(Devlin et al., 2015); ^3^(Sareen et al., 2013); ^4^(Naujock et al., 2016); ^5^(Pieri, 2003); ^6^(Kuo et al., 2004); ^7^(Kuo et al., 2005); ^8^(Bories et al., 2007); ^9^(van Zundert et al., 2008); ^10^(Quinlan et al., 2011); ^11^(Martin et al., 2013); ^12^(Leroy et al., 2014); ^13^(Pambo-Pambo et al., 2009); ^14^(Jiang et al., 2017); ^15^(Chang and Martin, 2011a); ^16^(Delestrée et al., 2014).

As direct access to LMN in humans is impeded for obvious reasons, a large part of studies addressing LMN electrophysiological alterations is conducted *in vitro*, employing induced pluripotent stem cell (IPSC)- derived MN from patient fibroblasts (Sareen et al., 2013; Wainger et al., 2014; Devlin et al., 2015; Naujock et al., 2016), cultured MN derived from spinal cord of early postnatal or embryonic mice (Pieri, 2003; Kuo et al., 2004; Kuo et al., 2005; Martin et al., 2013) or acutely isolated spinal cord/brain stem slices (Kuo et al., 2004; Bories et al., 2007; van Zundert et al., 2008; Pambo-Pambo et al., 2009; Quinlan et al., 2011; Martin et al., 2013; Leroy et al., 2014; Jiang et al., 2017; Figure 3 and Tables 3, 4). Thanks to recent advances in stem cell research, it is now possible to study the excitability and activity of MN derived from human patients’ fibroblasts through the generation of IPSC. These studies revealed that IPSC-derived MN from ALS patients are hyperexcitable and hyperactive in early cultures (2−6 weeks) (Wainger et al., 2014; Devlin et al., 2015), while they become hypoexcitable and hypoactive, as evidenced by a lower firing rate – input (F-I) gain and reduced spontaneous activity compared to control MN, when maintained longer (7−10 weeks old cultures) (Sareen et al., 2013; Zhang et al., 2013; Devlin et al., 2015; Naujock et al., 2016; Figure 3). The discrepancy between studies could potentially be explained by differences in the proportion of mature MN in cultures, as features like repetitive firing requires complete functional maturation (Devlin et al., 2015). Another important point to take into account is that while the majority of these cultures consist of neurons (80%), only ∼50% of which are classified as MNs while rest are potentially spinal interneurons (Devlin et al., 2015). Mirroring *early* hyperexcitability observed in rodent and human UMN as well as IPSC-derived LMN, cultured LMN derived from spinal cord of embryonic or neonatal SOD1^G93A^ tg mice were also shown to be hyperexcitable as seen in an increased firing frequency upon current injection (Pieri, 2003), increased maximum firing rate and F-I gain (Kuo et al., 2004), increased persistent Na^+^ current and decreased spiking threshold (Kuo et al., 2005; Figure 3). Similarly, hyperexcitability was also reported in slice culture studies obtained from embryonic/neonatal SOD1^G93A^ or SOD1^G93A–low^ tg mice (Figure 3). Whole-cell patch-clamp recordings made in lumbar/sacral spinal cord or brainstem slices demonstrated a more depolarized resting membrane potential and lower rheobase (Pambo-Pambo et al., 2009; Leroy et al., 2014), increased F-I gain (Kuo et al., 2004; Pambo-Pambo et al., 2009), firing frequency (Martin et al., 2013), persistent inward currents (Quinlan et al., 2011), and persistent Na^+^ current accompanied by enhanced spontaneous activity (van Zundert et al., 2008; Figure 3). However, Pambo-Pambo et al. (2009) demonstrated that the application of a different stimulation protocol (slow ramp current injection designed to test slow persistent currents), LMN from low copy SOD1^G93A^ as well as SOD1^G85R^ tg mice are hypoexcitable. These studies collectively report alterations of LMN excitability, observed mainly as initial hyperexcitability, which later changes into hypoexcitability. To investigate LMN in a more physiological setting, Delestrée et al. (2014) recorded from lumbar LMN in presymptomatic (P40−P50) SOD1^G93A^ anesthetized mice and found that their excitability was unchanged (current needed to trigger an AP), but their input conductance was increased (Figure 3). Furthermore, a substantial fraction of those had lost their ability to fire repetitively, arguing for the hypoexcitability of LMN in early stages *in vivo* (Delestrée et al., 2014). Seemingly at odds with these findings, are data pointing toward increased excitability of LMN (prolonged repetitive firing and higher frequency of spontaneous EPSPs) upon the stimulation of the dorsal root in acutely isolated sacral spinal cords obtained from presymptomatic − early symptomatic (P50−P90) SOD1^G93A^ tg mice (Jiang et al., 2017). Interestingly, based on pharmacological testing Jiang et al. propose that the observed changes in spontaneous depolarization originate at least in part from spinal network inputs rather than through the peripheral afferents (Jiang et al., 2017). Given the differences in sample preparation, recording technique, stimulation procedure and age of the animals, a decisive conclusion remains unattainable at the moment. Another important aspect to address, concerns the fact that LMN constitute distinct subtypes, which differ in their intrinsic excitability (Delestrée et al., 2014; Leroy et al., 2014; Martínez-Silva et al., 2018) and vulnerability (Pun et al., 2006; Hegedus et al., 2008; Saxena et al., 2009). As such, three main LMN subtypes can be distinguished. LMN innervating fast-fatigable (FF) extrafusal muscle fibers are the most vulnerable and degenerate early in the disease course. LMN innervating fast-resistant (FR) muscle fibers are less vulnerable and degenerate later in the disease, while those innervating slow (S) muscle fibers are resistant and persist until the end stage of the disease (Pun et al., 2006; Hegedus et al., 2008). In order to address whether the cell type specific vulnerability is reflected in different intrinsic properties of those subtypes, Martínez-Silva et al. performed intracellular recordings of LMN in deeply anesthetized mice *in vivo.* They discerned motor units (FF, FR, and S) based on their contractile properties by applying a pulse stimulation to the recorded LMN and measuring the force developed at the muscle tendon (Martínez-Silva et al., 2018). Investigations were performed in presymptomatic SOD1^G93A^ (P46−P60) and FUS^P525L^ tg mice (P30). They found that the most vulnerable FF type and a fraction of FR type LMN lost the ability to fire repetitively, thus became hypoexcitable, while S type LMN did not exhibit any changes in excitability (Martínez-Silva et al., 2018). The phenotype worsened with advancing disease, seen in an increase in the fraction of non-repetitively firing neurons in older FUS^P525L^ mice. These studies unraveled yet another level of complexity in ALS pathogenesis indicating large LMN subtype differences. Notably, S type LMN were shown to be hyperexcitable early postnatally (P6−P10), suggesting that hyperexcitability might even serve a neuroprotective role (Leroy et al., 2014). This notion is in concordance with an earlier publication, which demonstrated that treating low copy SOD1^G93A^ tg mice with a-amino-3-hydroxy-5-methyl-4-isoxazolepropionic acid (AMPA) agonist during the presymptomatic phase (P80), decreased misfolded SOD1 aggregates and ER stress and delayed muscle denervation by FR and S type LMN (Saxena et al., 2013). It also prevented the decline in muscle force, when administered slightly later (P145). Prolonged treatment (beginning ≤ P145) with AMPA receptor agonist even increased survival rates (Saxena et al., 2013). Notwithstanding, systemic application of the AMPA agonist cannot delineate the cellular/circuit mechanisms underlying the observed protective effect. To this end, LMN-selective, chemogenetic approaches were used to demonstrate that the effects were cell-autonomous (Saxena et al., 2009). Of note, the inverse experiment, that is blocking AMPA receptors by the application of 6-cyano-7-nitroquinoxaline-2,3-dione (CNQX), aggravated disease progression and shortened life span, thereby further substantiating the notion of neuroprotection by elevated excitation. These intriguing data contradict the dogma of glutamate-mediated excitotoxicity and the current therapeutic standards, which aim at reducing neuronal excitation (Bensimon et al., 1994). Despite not being fully conclusive, these findings collectively argue for more complex alterations of LMN excitability that are likely LMN subtype − and disease stage – dependent, which could explain why increased activation is protective for some while detrimental for others. Taken together, while findings of UMN excitability are more consistent, LMN excitability/activity in ALS warrants further scrutiny.

**TABLE 3:** Excitability related alterations of lower motor neurons (LMN) in rodent ALS models.

**Disease stage**	**ALS model**	**Age**	**Method of investigation**	**Finding**	**References**
Presymptomatic	SOD1^G93A^	neonatal	whole-cell patch-clamp recordings MNs cultured from spinal cord	hyperexcitability: firing frequency **↑**	(Pieri, 2003)
		neonatal and embryonic	intracellular recordings in neonatal organotypic spinal cord slice cultures	hyperexcitability: F-I gain **↑**, maximum firing rate **↑**	(Kuo et al., 2004)
			patch-clamp recordings in embryonic primary MN culture	hyperexcitability: F-I gain **↑**	
		embryonic	whole-cell patch-clamp recordings of MNs cultured from spinal cord	hyperexcitability: persistent Na^+^ currents **↑**, spiking threshold **↓**	(Kuo et al., 2005)
		P4−P10	whole-cell patch-clamp recordings in brainstem slices	hyperexcitability: persistent Na^+^ currents **↑**, spontaneous activity **↑**	(van Zundert et al., 2008)
		P0−P12	whole-cell patch-clamp recordings in isolated lumbar and sacral spinal cord	hyperexcitability: persistent inward currents **↑**	(Quinlan et al., 2011)
		E17.5	whole-cell patch-clamp recordings in isolated lumbar spinal cord	hyperexcitability: firing frequency **↑**	(Martin et al., 2013)
		P6−P10	whole-cell patch-clamp recordings in lumbar spinal cord, current stimulation of the ventral rootlet	S-type LMN hyperexcitability: rheobase **↓**, spiking threshold **↓**	(Leroy et al., 2014)
		P40−P50	intracellular recordings of LMNs in sacral spinal cord	normoexcitability: F-I gain **↔**, input conductance**↑**	(Delestrée et al., 2014)
		P34−P82	current clamp recordings of lumbar LMNs in anesthetized mice *in vivo*	normoexcitability: F-I gain **↔**, input conductance **↑** hypoexcitability: repetitive firing **↓**	
		P50−P90	intracellular recordings of LMNs and ventral root of isolated sacral spinal cord	hyperexcitability: repetitive firing **↑**	(Jiang et al., 2017)
		E12−P14	whole-cell patch-clamp recordings of MNs cultured from spinal cord	inhibition **↓**: glycinergic currents **↓**, glycinergic mIPSCs **↓**	(Chang and Martin, 2011a)
	SOD1^G93A^ and FUS^P525L^	P46−P60	current clamp recordings of lumbar LMNs in anesthetized mice *in vivo*	FF- and FR-type LMN hypoexcitability: repetitive firing **↓**	(Martínez-Silva et al., 2018)
	SOD1^G85R^	P6−P10	Intracellular recordings in isolated lumbar spinal cord	hypoexcitability: input resistance **↓**	(Bories et al., 2007)
		P6−P10	whole-cell patch-clamp recordings in isolated lumbar spinal cord	hypoexcitability: F-I gain to ramp current **↓**	(Pambo-Pambo et al., 2009)

*F-I, frequency-current; FUS, fused in sarcoma; LMN, lower motor neuron; mIPSCs, miniature inhibitory postsynaptic currents; MN, motor neuron; RMP, resting membrane potential; SOD1, superoxide dismutase 1.*

**TABLE 4:** Excitability changes detected on ALS patient IPSC-derived MN.

**ALS model**	**Age**	**Method of investigation**	**Finding**	**References**
fALS (*SOD1*^A4V, D90A, G85S^, *FUS*^MGH5b,H517Q^, *C9orf72*)	4 WIV	multielectrode array and whole-cell patch-clamp recordings in IPSC- derived MNs	hyperexcitability and hyperactivity: spontaneous activity **↑**, total/mean firing rate **↑** (*C9orf72*), DR K^+^ peak currents **↓** (*SOD1*^A4V^)	(Wainger et al., 2014)
fALS (*C9orf72*)	9−11 WIV	whole-cell patch-clamp recordings in IPSC-derived MNs	hypoexcitability: F-I gain **↓**, repetitive firing **↓**	(Sareen et al., 2013)
fALS (*TDP-43* ^M337V^ or *C9orf72*)	2−6 WIV		early hyperexcitability: F-I gain **↑**	(Devlin et al., 2015)
	7−10 WIV		switch to late hypoexcitability: single/adaptive/repetitive firing **↓**, synaptic activity **↓**, persistent VG K^+^ peak currents **↓**, FI Na^+^ peak currents **↓**	
fALS (SOD1 ^D90A, R115G^ *FUS*^R521L,R521C^, ^R495QfsX527^)	3−4 WIV		normoexcitability: single/repetitive firings **↔**, spontaneous activity **↔**, VG ion channel currents**↔**	(Naujock et al., 2016)
			early hyperexcitability: synaptic activity **↑**	
	7−10 WIV		switch to late hypoexcitability at ∼7−10 WIV in fALS cultures: repetitive firing **↓**, membrane capacitance **↓**, FI and DR K^+^ peak currents **↑** (FUS only), Na^+^ peak currents **↓**	
	13−14 WIV		hypoexcitability: synaptic activity **↓**	
fALS (*TDP-43*^A90V^)	4−5 WIV		hypoactivity: spontaneous activity **↓**	(Zhang et al., 2013)

**C9orf72*, chromosome 9 open reading frame 72 gene; fALS, familial ALS; sALS, sporadic ALS, sALS; DR, delayed-rectifying; FI, fast -inactivating; F-I, frequency-current; *FUS*, fused in sarcoma gene; RMP, resting membrane potential; *SOD1*, superoxide dismutase 1 gene; *TDP-43*, TAR DNA-binding protein 43; tg, transgenic; VG, voltage-gated; WIV, weeks *in vitro*.*

## The Role of Circuit Elements in Motor Neuron Excitability and Degeneration in ALS

Despite ample evidence indicating changes in intrinsic excitability of motor neurons (likely via cell autonomous mechanisms), there is also data arguing for altered synaptic inputs (non-cell autonomous processes) (Fogarty et al., 2015; Jiang et al., 2017). As neuronal activity hinges on both parameters, we will summarize the current knowledge of sources providing synaptic inputs and how these might be affected by disease pathology. To obtain a more complete picture of these mechanisms, we will first delineate the cortical and spinal cord circuitries facilitating and regulating UMN and LMN activity.

### Cortical Circuits and Drivers of Cortical Neurodegeneration in ALS

Upper motor neurons (a.k.a. Betz cells in humans) are PN that reside in cortical layer VB. They receive local excitatory input from intratelencephalic neurons (IT) within M1. IT neurons constitute neurons that project transcallosally (corticocortical, CC) and those projecting to the striatum (corticostriatal, CStr). UMN largely receive input from upstream layer II/III IT (CC) and intralaminar input within layer V, from both layer VA (CC, CStr) and VB (UMN, also referred to as pyramidal tract neurons, PT) (Figure 1). Remote input to UMN (PT) is provided by the contralateral motor cortex, secondary motor cortex, somatosensory cortices, sensory and motor thalamus, parietal − and frontal cortex (Anderson et al., 2010; Hooks et al., 2013; Shepherd, 2013; Yamawaki and Shepherd, 2015; Commisso et al., 2018; Figure 1). UMN (PT) are highly multiprojectional, known to innervate multiple targets through collaterals, branching off from the main axon that is projecting to its caudal destination in the spinal cord or brain stem (Shepherd, 2013; Figure 1). In addition to excitatory input, GABAergic interneurons provide inhibition within the local microcircuitry and across brain areas to modulate UMN activity (Isaacson and Scanziani, 2011; Tatti et al., 2017; Swanson and Maffei, 2019). Interneurons can be classified based on their morphology, physiological properties, postsynaptic target(s) and surface markers expressed (Markram et al., 2004; Tremblay et al., 2016). Three major, largely non-overlapping GABAergic interneuron subtypes in rodents and humans are parvalbumin (PV), somatostatin (SST), and ionotropic serotonin receptor 5-HT3a expressing interneurons [majority of which express vasoactive intestinal peptide (VIP)] (Rudy et al., 2011; Tremblay et al., 2016; Wood et al., 2017; Figure 1). Together they constitute 10−20% of the cortical neuronal population (Nigro et al., 2018; Swanson and Maffei, 2019). PV-expressing cells comprise ∼40% of the cortical GABAergic population and are known to be fast-spiking, with low input resistance, providing strong inhibition on PN (Bartos and Elgueta, 2012; Tremblay et al., 2016; Safari et al., 2017; Wood et al., 2017; Yu et al., 2019). They are typically basket cells, which synaptically target the soma and proximal dendrites of PN (Bartos and Elgueta, 2012; Safari et al., 2017; Veres et al., 2017). The second most common type are SST-expressing interneurons (30% of all interneurons), most of which are Martinotti cells that target the dendrites of PN (Nigro et al., 2018). Their somata are distributed throughout layers II to VI, but are most abundant in layer V (Scheyltjens and Arckens, 2016; Nigro et al., 2018). The remaining less well-characterized 5-HT3a subtype composes 30% of the interneuronal population (Lee et al., 2010; Rudy et al., 2011). Vasoactive intestinal polypeptide (VIP)-expressing interneurons are the most commonly seen within this subtype. Notably, VIPs mainly serve a disinhibitory role within the network as they inhibit SST- and PV-expressing interneurons (Lee et al., 2013; Yu et al., 2019). These excitatory and inhibitory inputs to UMN are fine-tuned, based on the behavioral context and attentional state, by multiple neuromodulatory systems, such as dopamine (DA), norepinephrine (NE, also known as noradrenaline), serotonin (5-HT), histamine (HA) and acetylcholine (ACh) (Gu, 2002; Conner et al., 2010; Shepherd, 2013; Vitrac and Benoit-Marand, 2017; Figure 1). Together, these different circuit elements are involved in directing UMN activity by regulating synaptic inputs. Functional deficits of one of these elements or compromised connectivity within the network can affect activity levels of UMN and impair information processing of UMN. Non-cell-autonomous processes have been shown to contribute to the reported cortical hyperexcitability. As such neurons and glia cells that shape and regulate the activity of MN need to be considered in the pathogenesis as well. The three main putative non-cell autonomous sources (excluding glia cells, which will be addressed in a separate paragraph), causing increased MN excitation, are thus: (1) increased excitatory input, (2) decreased inhibition, and (3) reduced neuromodulation. What is the evidence for a potential contribution of those factors?

#### Increased Excitatory Input

Electrophysiological recordings of LV PN in mouse models of ALS (SOD1^G93A^, TDP-43^Q331K^) demonstrated increased synaptic excitation, occurring already in early pre-symptomatic stages (Fogarty et al., 2016a, b; Saba et al., 2016). These electrophysiological changes were accompanied by structural changes seen in a lower cell complexity (reduction of dendritic arbor length) and a reduction of spines (sites of excitatory input) on apical and basal dendrites in the SOD1^G93A^ model, but an increase in spine density in the TDP-43 model (Fogarty et al., 2015, 2016a,b). Furthermore, an increase in the expression of vesicular glutamate transporter VGLU2 has been shown selectively in M1 of presymptomatic SOD1^G93A^ tg mice (Saba et al., 2016). This finding strongly indicates that presynaptic input to M1, including UMN, is increased either quantitatively or qualitatively. The source or cellular origin of this increased input, however, remains elusive thus far. Notably, a recent publication argues for aberrant connectivity and thus increased synaptic input to M1 provided by S1 and contralateral M2 already in juvenile (very early presymptomatic) SOD1^G93A^ tg mice (Commisso et al., 2018). This aberrant connectivity aggravated with disease progression, resulting in larger input from areas, such as the thalamus, contralateral M1, auditory cortex and the caudoputamen in later disease stages (Commisso et al., 2018). Of note, hyperconnectivity is also observed in ALS patients, thus providing a possible mechanism of cortical hyperexcitability (Commisso et al., 2018).

#### Compromised Inhibition

Increased excitation or a shift in the balance between excitation/inhibition (E/I) can also result from defective inhibition. Indeed, functional clinical studies employing TMS indicate that cortical hyperexcitability is at least in part based on a reduction of cortical inhibition (Prout and Eisen, 1994; Zanette et al., 2002; Vucic et al., 2008; Menon et al., 2015; Cengiz et al., 2019). Moreover, a reduction in GABA levels was found in motor cortex of ALS patients using proton magnetic resonance spectroscopy (MRS) (Foerster et al., 2012). This effect was only partially rescued by treatment with Riluzole (Foerster et al., 2012). Along these lines, an altered molecular composition of the GABA_A_ receptor has been shown, evidenced by a reduction of α1-subunit mRNA and an increase in the β1-subunit mRNA levels in postmortem motor cortex, which could indicate altered receptor function (Petri et al., 2003). Mouse models of the disease have further substantiated the notion of compromised inhibition in ALS pathophysiology. Whole-cell patch-clamp recordings of cultured interneurons from embryonic Gad67-GFP:SOD1^G93A^ mice revealed that interneurons (subtype not specified) were morphologically less complex and less excitable (Clark et al., 2018). Subtype-specific investigations have furthermore unraveled a selective impairment of different interneuron populations. The largest interneuron population in the cortex, PV-expressing interneurons, was shown to undergo disease-stage specific changes. While PV interneurons in presymptomatic (P26−P35) SOD1^G93A^ tg mice did not differ from WT controls, they turned hyperexcitable in symptomatic (P90−P129) mice (Kim et al., 2017), indicating a possible compensatory mechanism (Figure 1). The effects, however, might also be mutation specific, as in the TDP-43^A315T^ mouse model, PV interneurons were found to be hypoexcitable in presymptomatic mice and received more inhibitory synaptic input compared to the WT controls (Zhang et al., 2016). In the same model, the authors found a striking increase in the excitability of SST interneurons, which led them to propose a microcircuit model of UMN hyperexcitability, in which hyperexcitable SST interneurons depress PV interneurons, thereby releasing the break on UMN (Zhang et al., 2016; Figure 1). In addition to structural and electrophysiological changes, there are also contradictory findings regarding interneuron subtype-specific density in motor cortex. While the density of PV, SST and VIP interneurons remain unchanged in SOD1^G93A^ mice (Özdinler et al., 2011; Clark et al., 2017), there is a decrease in PV and SST density in Wobbler mice (Nieto-Gonzalez et al., 2011). Remarkably, a SOD1 zebrafish model of the disease even suggests that interneuron dysfunction precedes MN degeneration (McGown et al., 2013). It is relevant to stress that also interneurons express glutamatergic synapses, some of which even carry AMPA receptors lacking the GluA2 subunit, which renders them highly Ca^2+^ permeable akin to MN (Akgul and McBain, 2016), thus potentially also putting interneurons at risk of receiving large Ca^2+^ influx. Most importantly, one needs to acknowledge that a global anti-glutamatergic treatment will also affect the excitability and activity-dependent recruitment of inhibitory neurons, which could further exacerbate a potential inhibitory deficit. Though these are all intriguing novel aspects of cortical hyperexcitability, it remains to be clarified whether these findings are generalizable to other forms/mutations of the disease, whether the effect is disease-stage dependent and of course whether similar cell type specific changes are at play in human patients.

#### Impaired Neuromodulation

As neuromodulation further tunes neuronal activity, it could also modify E/I balance in M1. In ALS patients, there are data demonstrating a dopaminergic as well as a serotonergic deficit (Takahashi et al., 1993; Borasio et al., 1998; Sandyk, 2006; Vermeiren et al., 2018). Serotonin exerts amongst others inhibitory control of glutamatergic release via 5-HT_1__B_ presynaptic receptors (Muramatsu et al., 1998), the lack of which could contribute to excitotoxicity. Moreover, ALS patients have increased levels of norepinephrine in blood and the cerebrospinal fluid (CSF) (Ziegler et al., 1980), which could synergize with glutamate and increase neuronal excitation (Mather et al., 2016). For a more detailed description on the impact of altered neuromodulation in motor cortex in ALS see Brunet et al. (2020). Together, these studies emphasize that circuit elements other than the mainly affected UMN are strongly altered likely already early in the disease. Whether these precede or follow UMN excitability changes remains to be resolved. Therefore, non-cell autonomous mechanisms warrant more attention and further investigation.

### Spinal Circuit Elements Contributing to LMN Degeneration

In analogy to evidence indicating a casual involvement of circuit mechanisms in the degeneration of UMN, also LMN degeneration is likely a result of more complex alterations within spinal circuits. The majority of inputs LMN receive are provided by descending tracts from supraspinal regions e.g., cortex and brainstem. In humans, UMN-LMN connections are mainly monosynaptic, while in rodents this connection is polysynaptic, involving interposed interneurons (Lemon, 2008). Nevertheless, LMN activity is regulated by spinal interneurons in all mammals (Bikoff et al., 2016; Bikoff, 2019). For instance, the execution of a motor command is tightly controlled via class I interneurons (Ia-IN and Ib-IN, inhibitory: glycinergic), Renshaw cells (inhibitory: glycinergic/GABAergic), and class II interneurons (II-IN, excitatory: glutamatergic/cholinergic) (Côté et al., 2018; Figure 2). While class II-IN are the sole source of cholinergic inputs (Rozani et al., 2019), they are not the only neuromodulatory input LMN receive (Heckman et al., 2003). Descending tracts primarily from brainstem nuclei modulate LMN excitability via noradrenergic (from locus coeruleus) and rich serotonergic (from raphe nucleus) inputs, mainly facilitating their excitation (Heckman et al., 2003; Bruinstroop et al., 2012; Figure 2). Noradrenergic inputs project to the ventral horn (Jones and Yang, 1985; Bruinstroop et al., 2012), where multiple noradrenergic receptors are expressed, however, whether they form a direct monosynaptic connection to LMN is not clear (Smith et al., 1995, 1999; Day et al., 1997; Mizukami, 2004; Tartas et al., 2010; Figure 2). Furthermore, descending serotonergic inputs form both monosynaptic synapses with LMN (Sławińska and Jordan, 2019), as well as indirect synapses via spinal interneurons, comprising the central pattern generator (CPG). CPGs are neural networks in the spinal cord that generate rhythmic movements, which are largely independent of descending inputs from higher motor areas and sensory inputs from sensory afferents (Grillner, 2003). Spinal interneurons are proposed to provide their input to CPGs or form part of the network, which are regulated by neuromodulatory inputs (Gerasimenko et al., 2016; Laliberte et al., 2019). Moreover, LMN receive proprioceptive feedback from muscles via three major sensory afferent subtypes, namely Ia, Ib and II afferents (excitatory: glutamatergic) (Brown, 1981; Figure 2). An additional spinal interneuron population, presynaptic inhibitory interneurons (PI-IN, inhibitory: GABAergic), synapsing on Ia afferent terminals also tune final sensory input on the LMN (Stein, 1995; Fink et al., 2014; Figure 2). Furthermore, LMN regulate their own activity by activating Renshaw cells, which in turn inhibit LMN, a phenomenon called recurrent inhibition (Eccles et al., 1954; Cullheim et al., 1977; Nishimaru et al., 2005; Figure 2).

#### Excitatory Inputs

Excitatory glutamatergic input to the ventral horn is provided mainly by descending projections from motor cortex (corticospinal tract, UMN), pontine and medulla (reticulospinal tract, commissural interneuron), sensory afferents and partially by excitatory II-IN and LMN-Renshaw cell synapses (Brownstone and Bui, 2010; Côté et al., 2018; Figure 2). There have been reports of affected excitatory input to LMN in ALS (Menon et al., 2019; Seki et al., 2019). Modified excitatory input is also recapitulated in mouse models of ALS. Genetic reduction of vesicular-glutamate transporter 2 (VGLUT2) expression in SOD1^G93A^ tg mice reduced motor neuron loss in lumbar spinal cord and brainstem, but had no impact on disease onset or life span, suggesting a partial rescue of degenerative processes (Wootz et al., 2010). Another main glutamatergic input is provided by proprioceptive afferents, which also seem to be affected in the disease. Seki et al. (2019) have reported the occurrence of irregular action potential bursts selectively in Ia proprioceptive sensory neurons located in the brainstem of presymptomatic (P8−P14) SOD1^G93A^ tg mice. To address a potential impact of impaired sensory input on LMN function and health, Lalancette-Hebert et al. eliminated Ia inputs on LMN. To this end, they crossed SOD1^G93A^ tg mice with the Egr3^KO^ (early growth response 3 knockout) mutant mouse, which develops muscle spindle degeneration and thus lack proper Ia sensory feedback. The reduction of excitatory inputs from Ia afferents in SOD1^G93A^/Egr3^KO^ double mutants slowed down LMN loss, but again did not alter disease progression (Lalancette-Hebert et al., 2016). However, when in addition genetically ablating γ-MNs, which innervate muscle spindles, disease onset was delayed and lifespan increased (Lalancette-Hebert et al., 2016). Thus, alterations of excitatory sensory inputs seem to negatively affect LMN health in the disease.

#### Compromised Inhibition

As in cortex, impaired inhibition is also a feature of circuit deficits in the spinal cord in ALS. In the early 90s, an abnormal reduction of recurrent inhibition was reported in spastic ALS patients (Raynor and Shefner, 1994) and decreased glycinergic receptor expression has been found (Hayashi et al., 1981; Whitehouse et al., 1983; Figure 2). Moreover, a reduction in glycine levels in the serum was shown (Malessa et al., 1991), while others demonstrated the opposite (Kostera-Pruszczyk et al., 2002). Mouse models recapitulated the finding of compromised inhibition as evidenced by a progressive loss of glycinergic synapses on LMN presymptomatically and of Renshaw cells during the early symptomatic phase in SOD1^G93A^ tg mice (Chang and Martin, 2009) and decreased glycine transporter 2 (GlyT2) and glutamic acid decarboxylase (GAD65/67) expression in the ventral horn of symptomatic low-copy SOD1^G93A^ tg mice (Hossaini et al., 2011; Figure 2). The loss of inhibitory spinal interneurons (primarily but not exclusively Renshaw cells) was apparent in the late symptomatic stage, whereas motor neuron loss was reported at earlier time point, suggesting that motor neuron degeneration may trigger interneuronal pathology (Hossaini et al., 2011). In line with these findings, another study also reported a reduction of the inhibitory (vesicular GABA transporter, VGAT)/excitatory (VGLUT2) synapse ratio of hypoglossal motor neurons occurring already at presymptomatic stages in the same mouse model (Sunico et al., 2011). Of note, the imbalance resulted from an increase in excitatory synapses and a decrease in inhibitory contacts (Sunico et al., 2011). Cardinal aspects were also confirmed in cell culture models, where a decrease in postsynaptic glycine receptor expression was found (Chang and Martin, 2011a, b). Moreover, whole-cell patch clamp recordings in motor neuron cultures obtained from embryonic SOD1^G93A^ tg mice revealed decreased glycine-induced currents and glycine receptor expression on LMN, whereas no change in GABA_A_-induced currents (Chang and Martin, 2011a). Whereas, Carunchio et al. described an increased affinity of the GABA_A_ receptor subtype, expressed on cultured embryonic MN from SOD1^G93A^ tg mice, indicating a potential compensatory effect in response to reduced glycinergic inhibition (Carunchio et al., 2008).

#### Sensory Circuits

Sensory systems are considered less or not affected in ALS (Kawamura et al., 1981). However, there is also evidence for compromised sensory feedback onto LMN, the role of which in ALS pathology currently warrants further scrutiny. Sensory feedback from muscles to LMN is provided by afferents of proprioceptive sensory neurons, whose somata are located in the dorsal root ganglion (DRG) (Figure 2). This sensory feedback is regulated by spinal interneurons (Ia, Ib, II, and PI-IN). Early studies have revealed peripheral sensory nerve pathology, seen as axonal atrophy and increased remyelination in ALS patients (Heads et al., 1991; Figure 2). Further studies in symptomatic patients revealed reduced sensory nerve response amplitudes and conduction velocities (Hammad et al., 2007; Pugdahl et al., 2007; Sangari et al., 2016) and impaired dorsal column integrity, even in the absence of sensory deficits (Cohen-Adad et al., 2013; Figure 2). Mouse models of the disease partially recapitulate these findings, e.g., the degeneration of dorsal root axons was detected in presymptomatic SOD1^G93A^ tg mice, without obvious sensory deficits (Guo et al., 2009; Sassone et al., 2016). Moreover, sensory neurons (in particular Ia afferents) are also susceptible to cellular stress, misfSOD1 accumulation and mitochondrial damage at early disease stages (Guo et al., 2009; Sábado et al., 2014; Vaughan et al., 2018).

#### Altered Neuromodulation

Neuromodulatory transmitters, such as acetylcholine, serotonin, dopamine or noradrenaline play a prominent role in the adjustment of excitatory inputs to LMN (Zagoraiou et al., 2009; Tartas et al., 2010; Sharples et al., 2014; Sławińska and Jordan, 2019). There is ample evidence indicating profound alterations of neuromodulators in ALS pathophysiology. For instance cholinergic input provided by C-boutons was decreased in sporadic ALS patients (Nagao et al., 1998; Figure 2). Moreover, a reduction in ChAT expression was observed both at the protein (Oda et al., 1995) and the mRNA level in spinal cord of ALS patients (Virgo et al., 1992). Concordantly, the expression of choline acetyltransferase (ChAT) in C-boutons and in cholinergic interneurons of presymptomatic SOD1^G93A^ tg mice was also reduced compared to wild-type mice (Casas et al., 2013; Figure 2). In contrast, Pullen and Athanasiou (2009) reported an increased LMN C-bouton coverage in both presymptomatic and late-symptomatic SOD1^G93A^ tg mice. This discrepancy could be due to a LMN subtype – specific modulation of cholinergic inputs. Indeed, a disease-associated enlargement of C-boutons was confined to FF subtype LMN in the SOD1^G93A^ mouse model during the presymptomatic phase, while it affected the majority of LMN as disease progressed (Saxena et al., 2013). In order to test the hypothesis that C-bouton enlargement served a compensatory and neuroprotective role, Saxena et al. used a pharmacological approach to modify neuronal excitability. As proposed, treatment with the AMPA antagonist CNQX caused an increase of C-bouton size, while the AMPA agonist caused a decrease in size of those synapses on LMN (Saxena et al., 2013). These findings demonstrate that enhancement of LMN excitability through cholinergic input could serve as a compensatory mechanism in response to LMN activity reduction (Saxena et al., 2013). Behavioral support for this notion is provided by a study, in which the silencing of these premotor cholinergic interneurons compromised the compensation of impaired locomotor behavior at a much younger age in SOD1^G93A^ tg mice (Landoni et al., 2019). Notably, restoring C-boutons number and function in presymptomatic SOD1^G93A^ tg mice by viral-mediated delivery of type III-Neuregulin-1 (a trophic factor regulating neurotransmission and synaptic plasticity) extended survival (Lasiene et al., 2016). Together, these findings highlight the relevance of compensatory cholinergic input for LMN function and health. In addition to cholinergic input, LMN are also regulated by serotonergic and noradrenergic inputs. Earlier reports demonstrated increased levels of serotonin and noradrenaline in the ventrolateral lumbar spinal cords obtained post-mortem from ALS patients (Bertel et al., 1991). Corroborating these findings, serotonergic boutons were shown to be substantially increased around LMN in presymptomatic (P50) low-copy SOD1^G93A^ tg mice (Saxena et al., 2013; Figure 2). In contrast, serotonergic neuron degeneration was reported in ALS patients and SOD1^G86R^ tg mice, contributing to spasticity in the latter (Dentel et al., 2012). While serotonin mainly modulates LMN activity positively, depending on the specific receptor subtypes expressed on MN, it can also inhibit neuronal activity (Perrier et al., 2013). In addition to LMN also spinal interneurons, sensory afferent terminals, and astrocytes are modulated by serotonin via a large set of different serotonergic receptors (Bardoni, 2019). Therefore, the net effect of serotonin on LMN is also dependent on the activation of the particular interneuron population (inhibitory/excitatory) and the receptor types expressed on those neurons (Ciranna, 2006), further complicating the interpretation of those findings with respect to ALS pathophysiology. Overall, the data strongly argues for a compensatory increase of facilitatory neuromodulatory inputs to LMN in ALS. Collectively, it can be argued that a more complex dysregulation of spinal circuitries accompanies and potentially triggers LMN dysfunction and degeneration in ALS. The underlying sequence of events establishing cause and consequence and the nature (i.e., compensatory or detrimental) of the observed alterations remains to be elucidated.

## Role of Glial Cells in Neuronal Degeneration and Excitability

**FIGURE 4:**
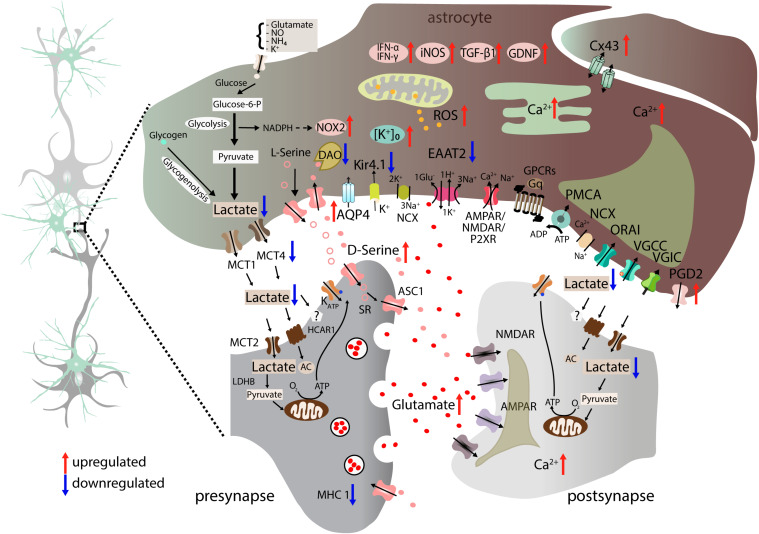
Altered neuron-astrocyte interaction in ALS. Astrocytes lose homeostatic function through multiple proposed molecular dysregulations. Astrocytic EAAT2 downregulation results in increased synaptic glutamate (red dots) and enhanced postsynaptic NMDAR and AMPAR activation. Moreover, astrocyte-mediated K^+^ clearance is disrupted due to downregulation of Kir4.1 and upregulation of co-localized AQP4 channels. Astrocyte-neuron lactate shuttle is impaired as a result of reduced MCT4 expression leading to lower extracellular lactate concentration (Ferraiuolo et al., 2011). Astrocytes gain toxic properties by generating factors such as ROS, NOX2, and iNOS (Marchetto et al., 2008), NOS or TGF-β1 (Endo and Yamanaka, 2015). In addition, they secrete proinflammatory cytokines IFN-γ and IFN-α/β (Aebischer et al., 2011) and upregulate PGD2 receptor expression. Glutamate-excitotoxicity is aggravated by increased levels of D-Serine (pink dots) due to reduced degradation caused by DAO downregulation in astrocytes. Furthermore, astrocytes also display Ca^2+^ dyshomeostasis in the cytosol and ER. Connectivity between astrocytes is altered by elevated expression of the gap junction protein Cx43. Blue arrows indicate downregulation, whereas red arrows show upregulation. AC, adenylyl cyclase; ADP, adenosine diphosphate; AMPAR, α-amino-3-hydroxy-5-methyl-4-isoxazolepropionic acid receptor; AQP4, aquaporin-4; ASC1, astrocytic transporter SLC7A10; ATM, ataxia telangiectasia mutated; Ca^2+^, calcium ion; Cx43, connexin 43; EAAT2, excitatory amino acid transporter 2; glucose-6-P, glucose-6-phophate; GDNF, glial cell-derived neurotrophic factor; GPCRs, G-protein-coupled receptors; Gq, q subunit of heterotrimeric G protein; HCAR1, hydroxycarboxylic acid receptor 1; IFN-α, interferon alpha; IFN-β, interferon beta; IFN- γ, interferon gamma; iNOS, inducible nitric oxide synthase; K^+^, potassium ion; Kir4.1, inward-rectifying potassium channel 4.1; LDHB, lactate dehydrogenase B; MCT1, monocarboxylate transporter 1; MCT2, monocarboxylate transporter 2; MCT4, monocarboxylate transporter 4; MHC1, major histocompatibility complex class I; NADPH, nicotinamide adenine dinucleotide phosphate hydrogen; NCX, sodium-calcium exchanger; NH_4_, ammonium; NMDAR, N-methyl-D-aspartate receptor; NO, nitric oxide; NOX2, NADPH oxidase 2; NOS, nitric oxide synthase; O_2_, oxygen; ORAI: Ca^2+^ release-activated Ca^2+^ channel protein 1; P2XR, ionotropic receptors; PDG2, prostaglandin D_2_ receptor; PMCA, plasma membrane Ca^2+^-ATPase; VGIC, voltage-gated ion channels.

Neuronal communication is strongly regulated and modulated by glial cells. In particular, astrocytes represent a vital component of neural circuits (Ben Achour and Pascual, 2012; Allen and Eroglu, 2017; Durkee and Araque, 2019). One of the core tasks is the effective clearance of glutamate from the synaptic cleft, which is a prerequisite for a spatiotemporal confined synaptic transmission and should protect neurons from detrimental “overstimulation” (Rothstein et al., 1996; Trotti et al., 1999). Since the early 2000s, glial cells have gained increasing attention as an integral part of ALS pathophysiology, but their role in the disease is still incompletely understood. Several molecular, genetic and transplantation studies argue for both a toxic gain-of-function (Nagai et al., 2007; Marchetto et al., 2008; Re et al., 2014; Rojas et al., 2015) as well as a loss-of-physiological function in astrocytes (Rothstein et al., 1995; Howland et al., 2002; Kaiser et al., 2006; Ferraiuolo et al., 2011; Bataveljic et al., 2012; Kawamata et al., 2014) in ALS (Figure 4 and Supplementary Table 3), which we will summarize here.

### Altered Astrocytic Function and Molecular Composition

#### Astrocytes as Regulators of Extracellular Glutamate

One of the earliest piece of evidence arguing for a causal involvement of astrocytes in ALS pathophysiology was the downregulation of the astrocytic glutamate transporter EAAT2 (a.k.a. GLT1 in rodents) in motor cortex and spinal cord of both familial and sporadic end-stage ALS patients (Rothstein et al., 1995; Figure 4 and Supplementary Table 3). Similar findings were made in mouse models of the disease both in spinal cord (SOD1^G85R^ and SOD1^G93A^ tg mice) (Bruijn et al., 1997; Howland et al., 2002; Wilson et al., 2003; Bendotti et al., 2008) and in motor cortex (ALS-PDC tg mice) (Wilson et al., 2003). This reduction was specific to the EAAT2, as the expression of the EAAT1 (GLAST) or the neuronal EAAT3 (EAAC-1) was not affected (Rothstein et al., 1995). The decrease in EAAT2 expression was suggestive of impaired glutamate uptake and was hence proposed to cause glutamate-mediated excitotoxicity on MN (Rothstein et al., 1995). This notion resonated within the field of neurodegeneration research, as astrocytic loss of EAAT2 expression was also observed in other neurodegenerative diseases, such as Alzheimer’s disease (Li et al., 1997; Jacob et al., 2007), Huntington’s disease (Arzberger et al., 1997; Shin et al., 2005; Faideau et al., 2010), and epilepsy (Binder and Steinhauser, 2006; Wetherington et al., 2008), arguing for a convergence of cellular mechanisms shared by these diseases. However, we still lack proof of a direct causal link between EAAT2 reduction and MN death. Manipulation of EAAT2 expression in rodent models of ALS thus far yielded mixed results. Heterozygous knock out of the glutamate transporter (SOD1^G93A^/GLT1^±^ tg mice) caused a faster disease progression and decreased survival slightly (Pardo et al., 2006). To address the question whether the reduction of GLT1 is sufficient to cause neuronal degeneration, in a seminal manuscript Rothstein et al. (1996) knocked out GLT1 in the CNS of rats by chronic intraventricular application of antisense oligonucleotides. As hypothesized, the consecutive decrease in GLT1 expression resulted in an increase in extracellular glutamate (30-fold increase – 16 μM), which was accompanied by ultrastructural changes such as cytoplasmic vacuolization, dilated Golgi apparatus and lamellated intracytoplasmic structures. Phenotypically, these mice exhibited progressive deficits in motor function, including ataxia, dystonia and hindlimb paralysis (Rothstein et al., 1996). One caveat of the study is that GLT1 levels in the spinal cord were not reduced upon the intraventricular infusion of antisense oligonucleotides. Thus, the observed motor deficits are likely mainly a result of damaged supraspinal motor areas at least in the initial phase. These studies confirm a toxic role of elevated glutamate levels. However, the resulting extracellular glutamate levels exceeded those seen in ALS patients (Plaitakis and Constantakakis, 1993; Shaw et al., 1995; Cid et al., 2003). Does overexpression of reuptake transporters mitigate disease symptoms? While this seems a conceivable approach, the hitherto available data is inconclusive. One early study reported a delay in muscle force loss, but no effect on disease onset or life span in SOD1^G93A^ tg mice crossed to a transgenic mouse line expressing the hEAAT2 under the GFAP promoter (Guo et al., 2003). Intriguingly, in a slice culture screening approach β-lactam antibiotics were identified to stimulate EAAT2 expression and were subsequently tested *in vivo* for its impact on disease progression in SOD1^G93A^ tg mice (Rothstein et al., 2005). Indeed, chronic treatment with Ceftriaxon, a β-lactam antibiotic, initiated at symptom onset (12 weeks) delayed neuronal loss, muscle weakening and increased mouse survival (Rothstein et al., 2005). These highly promising findings initiated a clinical trial probing the efficacy of Ceftriaxon to alleviate disease progression in ALS. Unfortunately, a stage-3 clinical study could not show any beneficial impact on symptom progression or survival in ALS patients, deeming the substance ineffective (Cudkowicz et al., 2014). Apart from a mere passive role provided by synaptic glutamate uptake, astrocytes have recently also been acknowledged as active partners regulating neurotransmission and synaptic function (reviewed by Chung et al., 2015). Amongst others, astrocytes were shown to instruct the composition of neuronal glutamate receptors, thereby determining neuronal vulnerability (Van Damme et al., 2007). To demonstrate this astrocytic property Van Damme et al. employed a MN-astrocyte co-culture system to investigate the interaction of both populations between two different rat strains. Both strains differed in the vulnerability of AMPA-mediated excitotoxicity due to differential expression of the GluR2 subunit of the AMPA receptor in MN (low level GluR2 AMPA receptors facilitate high Ca^2+^ permeability). Interchanging the astrocyte and MN population from both strains in the co-culture system revealed that astrocytes determine MN GluR2 abundance thereby regulating MN vulnerability (Van Damme et al., 2007). Importantly, the expression of SOD1^G93A^ abolished the capacity of astrocytes to up-regulate the GluR2 subunit and thus reduce the vulnerability of MN to excitotoxicity (Van Damme et al., 2007). Another interesting aspect of EAAT2 function is its potential detrimental role due to posttranslational modification. Earlier work has demonstrated that the EAAT2 can be cleaved by caspase 3 and the resulting sumoylated c-terminal fragment subsequently accumulates within astrocytes, conveying neurotoxic effects (Foran et al., 2014). A number of other posttranslational modifications of the EAAT2, such as palmitoylation, nitrosylation or ubiquitination have been identified (Peterson and Binder, 2019), but their putative impact on ALS pathophysiology remains to be clarified.

#### Astrocytes as Regulators of Extracellular Potassium

In addition to impaired glutamate re-uptake, disrupted astrocyte-mediated clearance of K^+^ (termed spatial K^+^ buffering) and H_2_O was suggested to promote neuronal hyperexcitability (Haj-Yasein et al., 2011; Florence et al., 2012; Devinsky et al., 2013; Bellot-Saez et al., 2017), and therefore could contribute to glutamate-mediated excitotoxicity (Figure 4 and Supplementary Table 3). Extracellular K^+^ is taken up into astrocytes via Na^+^/K^+^ ATPases (Hertz and Chen, 2016) and inward-rectifying Kir4.1 channels, the latter of which is also important for K^+^ re-release from astrocytes (Bay and Butt, 2012). Kir4.1 channels can co-localize with the water channel, aquaporin 4 (AQP4), indicating functional synergy (Nagelhus et al., 1999; Figure 4). Previously, a decrease in Kir4.1 expression was observed in the ventral horn of the spinal cord in pre-symptomatic SOD1^G93A^ mice (Kaiser et al., 2006; Figure 4). In addition, a reduction of Kir4.1 channel sensitivity and number, along with an increased expression of AQP4 was observed in both motor cortex and brain stem of symptomatic SOD1^G93A^ tg rats (Bataveljic et al., 2012). Corroborating these results, iPSC-derived astrocytes obtained from ALS patients with a SOD1 mutation also showed decreased Kir4.1 expression (Kelley et al., 2018). These findings indicate a lowered K^+^ buffering capacity of mutant astrocytes. Indeed, conditional knock out of Kir4.1 caused premature death, seizures and ataxia and was shown to cause membrane depolarization as well as an impairment of K^+^ and glutamate uptake by astrocytes (Djukic et al., 2007). To determine the pathological role of the reported Kir4.1 downregulation in ALS, Kelley et al. (2018) knocked out astrocytic Kir4.1 channels in spinal cord of SOD1^G93A^ tg mice, but found no further acceleration of MN loss. The pathophysiological relevance of Kir4.1 reduction in ALS thus remains unresolved.

#### Astrocytes as Providers of Energy

Lactate is one of the main energy substrates for neurons and is predominately produced in astrocytes from glucose or glycogen. Lactate has gained increasing attention in the modulation of neuronal excitability, plasticity and neuroprotection (Magistretti and Allaman, 2018). Notably, lactate generation in astrocytes hinges on extracellular glutamate, thus neuronal activity, which stimulates the uptake of glucose into astrocytes (Pellerin and Magistretti, 1994; Magistretti and Allaman, 2018). The astrocyte-neuron lactate shuttle (ANLS) model (Pellerin and Magistretti, 1994) proposes that astrocytes produce lactate and release it through transmembrane monocarboxylate transporters (MCTs, in particular MCT1 and MCT4), high-capacity cation channels and pannexins into the extracellular space, from where neurons take it up through transmembrane transport by MCT2 or G-protein coupled receptors [hydrocarboxylic acid receptor 1 (HCAR1) − reducing cAMP and thus reducing neuronal activity (Bozzo et al., 2013) and through a still unidentified Gs-coupled receptor] (Figure 4). Within the neuron, lactate is metabolized to pyruvate and NADH, stimulating a plethora of intracellular signaling cascades (Magistretti and Allaman, 2018; Figure 4). Lactate was suggested to serve a neuroprotective role against glutamate-mediated excitotoxicity (Magistretti and Allaman, 2018). Supporting this notion, treatment with L-lactate reduced the lesion size and neuronal death induced by high doses of glutamate (Ros et al., 2001). What is known about lactate and the ANLS in ALS? Cell culture and animal experiments indicate a lactate deficiency in ALS. Using *in vivo* ^1^H magnetic resonance spectroscopy (^1^H-MRS) approach Lei et al. (2019) observed a reduction of lactate in SOD1^G93A^ tg mice most prominently seen in motor cortex, starting at the early symptomatic phase in motor cortex and in the late symptomatic stage also in brainstem. In an astrocyte-MN co-culture system of tg astrocytes with WT MN, lactate was reduced, when compared to cultures containing WT astrocytes, indicating defects in the generation or release of lactate by astrocytes (Ferraiuolo et al., 2011; Madji Hounoum et al., 2017; Figure 4 and Supplementary Table 3). Treating those co-cultures in addition with glutamate further aggravated the reduction of lactate, along with a strong modification of diverse metabolic pathways (Madji Hounoum et al., 2017). Taken together, the aforementioned studies suggest a lactate deficiency in ALS, which could cause metabolic dysfunction and compromised neuroprotection.

### Toxic Gain of Function of Astrocytes

As opposed to the loss of physiological function, there is compelling *in vitro* and *in vivo* evidence for astrocytes secreting toxic factors causing MN degeneration and death in ALS (Nagai et al., 2007; Re et al., 2014). The majority of studies rely on the application of conditioned media derived from cultured astrocytes of transgenic mice or IPSC-derived from ALS patients (sALS and diverse fALS cases) or astrocyte-MN co-culture systems, which can cause dysfunction and/or death of cultured MN (Nagai et al., 2007; Re et al., 2014; Rojas et al., 2014; Zhao et al., 2020). In *in vivo* settings, in which conditioned media from cultured astrocytes expressing SOD1^G93A^ was infused into the spinal cord of rats, motor dysfunction was observed upon 8 days of infusion, accompanied by MN death and reactive astrogliosis (Ramirez-Jarquin et al., 2017). Transplantation studies, in which SOD1^G93A^ glial-restricted precursor cells (Papadeas et al., 2011) or astrocytes differentiated from patient derived IPSC (Chen et al., 2015) were injected into the spinal cord, revealed the development of motor deficits. However, the effect might be mutation-specific, as astrocytes carrying the TDP-43^A315T^ mutation did neither cause MN degeneration in co-culture nor upon transplantation into rat spinal cord *in vivo* (Haidet-Phillips et al., 2013). A corresponding tg rat model, based on the astrocyte-selective expression of the TDP-43^M337V^, on the other hand, developed a severe phenotype consisting of MN loss, progressive paralysis and premature death (P70−P80) (Tong et al., 2013). Notably, the inverse experiment, that is the focal transplantation of WT astrocytes (glial-restricted precursors) into the cervical spinal cord of SOD1^G93A^ tg rats, exerted protective effects, thus mitigating fore-limb motor deficits and the corresponding motor neuron loss, and even prolonged life span (Lepore et al., 2008). Surprisingly, a similar approach employing human glial-restricted progenitors transplanted into spinal cord of SOD1^G93A^ tg mice was ineffective (Lepore et al., 2011), potentially indicating species differences. Is the toxic effect conveyed by astrocytes mediated through changes in MN excitability? Again, the results are somewhat inconclusive: Conditioned media from cultured astrocytes expressing SOD1^G93A^ was sufficient to cause acute hyperexcitability on cultured MN as evidenced by increased persistent Na^+^ inward currents, repetitive firing and calcium transients followed by MN death days later (Fritz et al., 2013). On the other hand, co-culture of IPSC-derived astrocytes from *C9orf72* mutation carriers caused hypoexcitability and actual activity loss in MN due to impaired activity of voltage-activated Na^+^ and K^+^ currents (Zhao et al., 2020). What is known about those putative toxic factor(s) released by astrocytes? Although multiple molecular candidates were proposed, a final conclusive answer to that question is still pending. Obvious candidates, such as glutamate, TNFα or interleukin-1β, interleukin-6 and interferon-γ could readily be excluded (Nagai et al., 2007). While the identification of a single toxic molecule proves highly difficult, a number of pathways involved were successfully unraveled. As such, it has been shown that the detrimental effects of conditioned astrocytic media involved the activation of Na^+^ channels causing hyperexcitability, concomitant Ca^2+^ influx, mitochondrial damage and the generation of reactive oxygen species (ROS), triggering apoptosis (Rojas et al., 2014, 2015). Others also found evidence for an impact on astrocytic health, including increased levels of ROS and NADPH oxidase 2 (NOX2) within astrocytes derived from spinal cord and brain of SOD1^G93A^ and SOD1^G37R^ tg mice, respectively (Cassina et al., 2008; Marchetto et al., 2008; Figure 4). Incubating SOD1^G93A^ expressing astrocytes with antioxidants and nitric oxide synthase inhibitors, prevented MN loss, indicating that the release of potential toxic factors involves mitochondrial damage and ROS generation within astrocytes (Marchetto et al., 2008). Another candidate is the prostaglandin D2 (PGD2) receptor, which is upregulated in SOD1^G93A^ expressing astrocytes. Pharmacological blockade of the PGD2 receptor ameliorated the toxic affects conveyed by tg astrocytes onto WT MN (Di Giorgio et al., 2008), due to hitherto unknown mechanisms. Furthermore, the pro-inflammatory cytokine interferon-γ (IFN-γ) was found to play a role in astrocyte-mediated neurotoxicity. At odds with the initial study by Nagai et al. (2007) interferon signaling pathways, most prominently involving IFN-γ (Aebischer et al., 2011) and IFN-α/β (Wang et al., 2011) were shown to be upregulated in astrocytes of SOD1^G93A^ tg mice (Figure 4). Inhibition of IFN signaling by either knock-out of the IFNα receptor 1 or knock out of the LIGHT-LT-βR protein (downstream of IFN-γ)

prolonged survival of SOD1^G93A^ tg mice (Aebischer et al., 2011; Wang et al., 2011). The discrepancy between studies regarding the change in IFN- γ concentration was assigned to species differences (rat vs. mouse) and the source, MN were derived from (primary cell culture vs. embryonic stem cell derived). In addition to the molecules mentioned above, transforming growth factor β (TGF-β), a cytokine important for immune response regulation (Butovsky et al., 2014; Yamanaka and Komine, 2018) has also been implicated in astrocyte-mediated toxicity. In various mouse models of the disease (SOD1^G93A^, SOD1^G85R^, SOD1^G37R^) as well as in sALS patients, TGF-β1 expression was increased (Endo et al., 2015; Endo and Yamanaka, 2015) (Figure 4). Selective overexpression of TGF-β1 in astrocytes in the SOD1^G93A^ model (crosses of SOD1^G93A^ and GFAP-TGF-β1 tg mice) accelerated disease progress, while pharmacological inhibition of TGF-β signaling prolonged survival of SOD1^G93A^ tg mice (Endo et al., 2015). Yet another molecule suggested to be involved in astrocyte-triggered MN dysfunction is D-Serine. D-Serine is an endogenous co-activator of NMDA receptors (Mothet et al., 2000; Panatier et al., 2006), and thus a prime candidate molecule involved in the observed excitability changes in ALS. Notably, mutations in the gene encoding the D-amino acid oxidase (DAO), a D-Serine degrading enzyme, is causing fALS (Mitchell et al., 2010; Kondori et al., 2018) and D-Serine expression is increased in the spinal cord (mainly located to glia cells) of fALS and sALS patients, as well as in symptomatic SOD1^G93A^ tg mice (Sasabe et al., 2007; Figure 4). Genetic knock out of DAO causes MN degeneration and abnormal locomotor behavioral (but not a full ALS-like phenotype) (Sasabe et al., 2012). DAO activity was also markedly reduced in SOD1^G93A^ tg mice, particularly in the anterior column of the spinal cord, a region where descending motor tracts impinge on LMN, and could be localized to astrocytes (Figure 4). These data suggest that astrocytes actively contribute to increased D-Serine levels, which promotes glutamate-mediated excitotoxicity through NMDA receptors. This hypothesis, however, took a slight turn as a recent publication unraveled that L-Serine instead of D-Serine was released by astrocytes (Wolosker et al., 2016). L-Serine is converted into D-Serine within neurons by the serine racemase (SR) and consecutively released by antiporters in a non-vesicular-dependent manner (Wolosker et al., 2016; Figure 4). Released D-Serine can be taken up by neurons and astrocytes, that latter of which degrade D-Serine through the DAO (Wolosker et al., 2016). Despite evidence arguing against gliotransmission of D-Serine by astrocytes, major findings, indicating increased D-Serine activity, still hold true and underscore a potential causal involvement in ALS pathophysiology. Most studies to date view astrocytes in ALS as a homogenous population. However, there is evidence arguing for a prominent role of only a subset of these in ALS pathophysiology (Diaz-Amarilla et al., 2011; Jiménez-Riani et al., 2017; Liddelow et al., 2017). This notion first arose as astrocytes with an aberrant phenotype were detected in primary spinal cord cultures of SOD1^G93A^ tg rats (Diaz-Amarilla et al., 2011). Those astrocytes (termed AbAs) possessed a high proliferative capacity and their supernatant was even more toxic to motor neurons than that of mixed astrocyte cultures. Recent work now indicates that these neurotoxic reactive astrocytes (termed A1 astrocytes) in fact represent a common feature of CNS diseases (Liddelow et al., 2017). A1 astrocytes lose a number of physiological functions and instead gain neurotoxic properties. These highly intriguing recent findings even suggest a general causative role of reactive astrocytes in CNS disorders.

## Glutamate-Mediated Excitotoxicity

One of the central tenets in ALS research, the “glutamate-mediated excitotoxicity” (and also in other neurodegenerative diseases), posits that neuronal degeneration occurs due to excessive glutamatergic stimulation as a result of compromised glutamate uptake and/or increased presynaptic release (Heath and Shaw, 2002). How do recent findings of altered intrinsic excitability/activity of and synaptic inputs to MNs reconcile with this prevailing dogma? Being the main excitatory neurotransmitter in the CNS, glutamate is released from the presynapse upon AP firing and binds to high-affinity Ca^2+^ permeable ionotropic receptors, namely NMDA -, AMPA -, kainate -, and metabotropic glutamate receptors (Blanke and VanDongen, 2009; Wright and Vissel, 2012). Glutamate is subsequently removed from the synaptic cleft by Na^+^-dependent excitatory amino-acid transporters expressed on both neurons and astrocytes (EAATs in humans, and GLAST-1 and GLT1-1 in rodents) (O’Donovan et al., 2017) and recycled into the presynaptic terminal. When glutamate removal from the synaptic cleft is compromised, the extended presence or larger quantities of glutamate could cause an increased Ca^2+^ influx into the postsynapse, resulting in “hyperexcitation.” A prolonged increase in intracellular Ca^2+^ is suggested to exhaust the Ca^2+^ buffering capacity of the cell, provided by calcium binding proteins, the endoplasmic reticulum (ER) and mitochondria (Grosskreutz et al., 2010; Lautenschlaeger et al., 2012). Furthermore, a potential disruption of Ca^2+^ handling by the ER/mitochondria has been suggested to cause the generation of reactive oxygen species (ROS) (Heath and Shaw, 2002; Grosskreutz et al., 2010). Since vulnerable MNs express low levels of GABA_A_ and glycine receptors (Lorenzo et al., 2006), high levels of Ca^2+^-permeable GluR2-deficient AMPA receptors, but low levels of Ca^2+^ buffering proteins, such as parvalbumin and calbindin (Ince et al., 1993; Leal and Gomes, 2015), rendering them susceptible to excess neuronal stimulation and Ca^2+^ influx. Yet, what is the actual evidence for excess glutamate in ALS pathophysiology? In the early 90s, elevated levels of glutamate in the blood (Babu et al., 1998; Kostera-Pruszczyk et al., 2002) and CSF of ALS patients were reported (Rothstein et al., 1990). Others, however, could not confirm these findings (Perry et al., 1990), found only an increase of glutamate in the serum and not in the CSF of sALS patients (Kostera-Pruszczyk et al., 2002) or even observed decreased glutamate levels in the CSF of fALS patients (primarily SOD1 fALS) (Wuolikainen et al., 2011). Mechanistically, an impaired glutamate uptake had been proposed based on the observation of reduced GLT-1 expression in postmortem cortex and spinal cords obtained from ALS patients (Rothstein et al., 1995). But to what extent do glutamate levels in the CSF or serum reflect synaptic concentration? Under physiological conditions, the glutamate concentration is ∼20−50 μM in serum plasma and ∼0.2−4 μM in CSF (Perry et al., 1990; Kostera-Pruszczyk et al., 2002; Cid et al., 2003). In ALS patients a range from ∼40 to 100 μM in plasma and ∼6–10 μM in the CFS was observed (Perry et al., 1990; Rothstein et al., 1990; Plaitakis and Constantakakis, 1993; Shaw et al., 1995; Kostera-Pruszczyk et al., 2002; Cid et al., 2003). In order to probe a potential toxic effect on MN, Cid et al. tested glutamate levels reminiscent of CSF concentrations in ALS patients (∼5.8 μM) and healthy controls (<2.8 μM) on cultured MNs and reported MN death when treated with glutamate levels found in ALS patients (Cid et al., 2003). Furthermore, the toxicity conveyed by the CSF from ALS patients could be prevented by the application of AMPA and kainate receptor antagonists in cell culture (Couratier et al., 1993; Sen et al., 2005). Chronic delivery of 3 and 5 mM kainic acid (kainate and AMPA receptor agonist) via intrathecal pumps for 4 weeks resulted in MN death in the spinal cord of mice and rats (Sun et al., 2006; Blizzard et al., 2016). While the latter experiments seem to support the glutamate-mediated excitotoxicity hypothesis, it is important to realize that the concentrations applied are two orders of magnitude higher than the glutamate levels observed in the CSF of ALS patients. Notably, chronic non-selective pharmacological blockade of glutamate uptake transporters via osmotic minipumps in the rat spinal cord did not cause any LMN degeneration or motor deficits, although the extracellular glutamate levels were ∼4-5 μM, being 3−4 times higher than in the control group (Tovar-y-Romo et al., 2009). Additional *in vivo* studies reported a particular vulnerability of dorsal horn neurons upon chronic AMPA administration (Nakamura et al., 1994; Hirata et al., 1997) or combined blockade of glutamate reuptake transporters and glutamate (Hirata et al., 1997). However, the authors did not observe any degeneration in the ventral horn, where LMN are located (Hirata et al., 1997). These studies indicate that AMPA receptor activation, but not elevated extracellular glutamate *per se*, can drive MN degeneration. Indeed, Corona and Tapia (2004) showed that microdialysis of a glutamate transport inhibitor (for ∼1 hour) into the rat spinal cord does not cause MN loss, despite increased glutamate levels (∼6−7 μM). When they, however, enhanced AMPA receptor activation, by perfusing 6−12 mM AMPA (for ∼25 min), 90−100% of MNs were lost. These *in vivo* studies have one point in common: the application of high doses of glutamate agonists (mainly AMPA) that are orders of magnitude higher than the synaptic glutamate levels (Moussawi et al., 2011). Extracellular glutamate concentrations range from 0.02 to 30 μM. This wide range of concentrations reflects the variation of synaptic, perisynaptic and non-synaptic glutamate levels, which needs to be taken into account to understand the effect of increased extracellular glutamate. Under resting/baseline conditions, the extracellular glutamate concentration varies from 0.02 to 2.0 μM (Moussawi et al., 2011). This concentration can even drop to nanomolar levels as assessed in acute hippocampal slices (Herman and Jahr, 2007). *In vivo*, the synaptic glutamate levels can increase to millimolar levels during neurotransmission, returning to 0.02 μM in less than 10 ms via glial and neuronal reuptake (Dzubay and Jahr, 1999). Is there a link between hyperexcitability and glutamate excess? Assuming a reduction of glutamate reuptake was the initial driver of increased glutamate levels, enhanced excitatory synaptic input could be a consequence. However, homeostatic mechanisms are expected to cause a change in synaptic scaling and intrinsic excitability to compensate for increased excitatory drive (Turrigiano et al., 1998; Joseph and Turrigiano, 2017), in which case a reduction of excitability would occur. Is the observed hyperexcitability of MN thus unrelated to glutamate levels, and even further boosts glutamatergic release, causing a vicious cycle? Which factors could be involved in disabling homeostasis in MN? Or is hyperexcitability (at least convincingly demonstrated for motor cortex) rather a result of altered E/I balance within the network instead of a cell autonomous process? Is it possible that UMN and LMN degeneration are disparate processes caused by different mechanisms? The apparent discrepancy of excitability changes and proposed molecular alterations thus open up many questions. Taken together, findings on glutamate-mediated excitotoxicity described above are circumstantial due to a number of reasons including the following: (i) The actual synaptic glutamate exposure time and concentration under physiological conditions and in ALS patients is yet to be disclosed (Moussawi et al., 2011), thus hampering proper *in vitro* and *in vivo* modeling of this disease aspect. (ii) Increased levels of glutamate in CSF or serum do not necessarily reflect the synaptic concentrations a MN is exposed to, mainly due to very fast glial clearance mechanisms. (iii) Cell type-specific effects of excess glutamate need to be addressed *in vivo*. Inhibitory interneurons in cortex and spinal cord also express glutamate receptors, some of which even highly Ca^2+^-permeable AMPA receptors lacking the GluA2 subunit (Akgul and McBain, 2016), thus potentially also putting interneurons at risk in ALS. Transcriptomic and proteomic datasets of these distinct populations have only recently been gathered, leaving many aspects of neuronal activity regulations still unanswered (D’Erchia et al., 2017; Maniatis et al., 2019; Marques et al., 2019). (iv) Homeostatic and compensatory mechanisms or the failure thereof have not been addressed yet. Healthy neurons are endowed with the necessary molecular machinery to compensate for altered input by adjusting their intrinsic excitability or synaptic strength in order to maintain output, a phenomenon called homeostatic plasticity (Hengen et al., 2013, 2016). One of the molecules involved in sensing altered neuronal activity is the Calcium/Calmodulin-dependent protein kinase type IV (CaMKIV), which senses Ca^2+^ influx in response to AP firing of a neuron (Hengen et al., 2013; Joseph and Turrigiano, 2017). Importantly, many CNS disorders are accompanied by an increase in intracellular Ca^2+^, including MN in ALS (Leal and Gomes, 2015). Pathological Ca^2+^ elevations could thus undermine the activity sensing machinery of a neuron. However, if this was the case one would expect to find hypoexcitability in affected neurons, which is in fact the case for LMN when recorded *in vivo* (Leroy et al., 2014; Martínez-Silva et al., 2018). While this might be a too simplistic model to explain MN degeneration in ALS, it yet emphasized the question as to how and why altered excitability occurs in the first place, indicating a failure of the homeostatic machinery.

## Concluding Remarks

**FIGURE 5:**
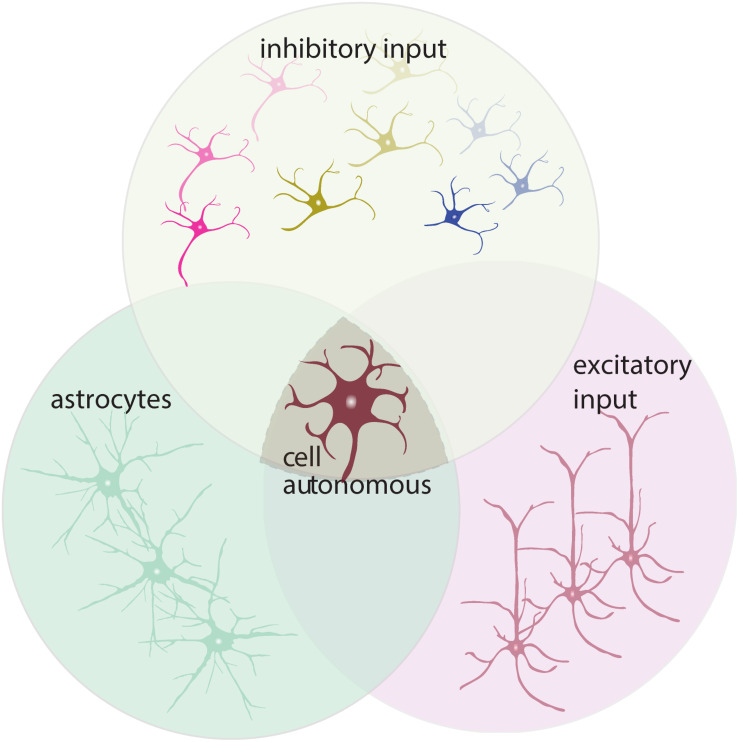
Interaction and synergy of proposed cell-autonomous vs. non-cell-autonomous mechanisms contributing to MN degeneration. Schematic depicting interdependency of proposed mechanisms. Cell-autonomous mechanisms refer to changes that take place within the affected MN, e.g., altered receptor expression. Non-cell-autonomous mechanisms refer to changes occurring on cell types other than MN, causally involved in the degenerative process, e.g., increased excitation (purple), lack of inhibition (yellow) and dysfunctional glutamate-reuptake system involving astrocytes (green).

We here summarized the current knowledge regarding structural and functional deficits of different elements of cortical and spinal cord motor circuitries in ALS. In summary, there is compelling evidence that motor cortex in ALS is hyperexcitable, the underlying mechanisms of which remain to be elucidated, but likely involve increased excitation and decreased inhibition simultaneously. The current data available for LMN and spinal circuits, however, appear less conclusive, but most recent *in vivo* recordings along with IPSC-derived MN approaches rather argue for a hypoexcitability of LMN. Changes in neuronal excitability and/or activity of different cell types and the consecutive impairment of neural circuit function have been reported in a number of CNS disorders, such as ALS-FTD (Zhang et al., 2016), Alzheimer’s disease (Busche et al., 2008; Grienberger et al., 2012; Liebscher et al., 2016; Schmid et al., 2016), Parkinson’s disease (Taverna et al., 2008), spinal muscular atrophy (Mentis et al., 2011), Huntington’s disease (Burgold et al., 2019), multiple sclerosis (Ellwardt et al., 2018), schizophrenia (Mukherjee et al., 2019), and glioblastoma (Venkatesh et al., 2019). What is the basis and what are the consequences of altered neuronal excitability? There are likely different mechanisms at play in the different diseases. In this review we have delineated a plethora of molecular and cellular mechanisms suggested to play a role (Figure 5). To conclusively answer that question, it is important to also acknowledge that “no neuron is an island.” In other words, neurons are embedded within complex local and global circuits, in which their activity is in addition to cell autonomous processes, regulated by numerous feedforward and feedback loops (Figure 5). Consequently, even “pure” cell autonomous defects will very likely leave a mark on the connected neurons. As changes in structure, function (excitability/activity/response properties) and connectivity develop gradually over time (as far as we know) and are paralleled by compensatory or even maladaptive processes of diverse network elements, the identification of the initial perturbation remains challenging. Are excitability changes relevant after all or rather represent an epiphenomenon? The current data convincingly shows that excitability and activity alterations of diverse neuronal subtypes are linked to markers of degeneration, such as the accumulation of intracellular protein aggregates and ER stress, and importantly that the manipulation of neuronal excitability can exert pronounced beneficial effects including a prolongation of life span (Saxena et al., 2013; Zhang et al., 2016). But these studies also highlight that the excitability modulation needs to be cell type and disease stage dependent, which might explain the failure of hitherto clinical trials. The enormous challenge for the future will thus be to piece together the jigsaw puzzle of the wealth of factors and processes identified thus far and determine their causal relevance and sequence of events in human ALS pathophysiology. A successful therapeutic strategy, in particular so as patients are almost exclusively diagnosed, when major tissue damage has occurred, will likely have to consist of a combinatorial treatment taking into account cell type-, brain area- and disease stage- specific alterations.

## Author Contributions

All authors wrote and edited the manuscript.

## Conflict of Interest

The authors declare that the research was conducted in the absence of any commercial or financial relationships that could be construed as a potential conflict of interest.
