# Supplementary material for: Exciting Complexity: The Role of Motor Circuit Elements in ALS Pathophysiology
Source: Front Neurosci. 2020 Jun 17;14:573. doi: 10.3389/fnins.2020.00573 (PMC7311855; doi:10.3389/fnins.2020.00573)
Supplement: Supplementary file 1 [file Table_1.pdf]

Supplementary Table 1. Key characteristics of commonly used mouse models of ALS (excluding C9orf72 repeat expansion models). Abbreviations: Alsin^{KO}, Alsin knockout; FUS, fused in sarcoma; hPFN1, human profilin 1; LMN, lower motor neurons; n.a., not applicable; n.s., not specified; SOD1, superoxide dismutase 1; TDP-43, TAR DNA-binding protein 43; UCHL1 null, ubiquitin carboxyterminal hydrolase 1 gene knockout; UMN, upper motor neurons; +, present; -, absent.

model	gene copy number	disease onset (months)	life-span (months)	motor phenotype (tremor, muscle weakness)	paralysis (at the humane end-stage)	UMN loss	UMN deficits	LMN loss	LMN deficits	references
SOD1^{G93A}	25	3	4	+	+	+	+	+	+	(Gurney et al., 1994; Chiu et al., 1995; Özdinler et al., 2011)
SOD1$^{G93A\text{-}low}$	8-10	4-5	8-8.5	+	+	n.s.	n.s.	+	+	(Acevedo-Arozena et al., 2011)
SOD1^{G85R}	0.2-1	8	8.5	+	+	n.s.	n.s.	+	+	(Bruijn et al., 1997)
SOD1^{G86R}	high/low	3-4	4	+	+	n.s.	n.s.	+	+	(Ripps et al., 1995)
SOD1^{G37R}	4-12	3-6	7	+	+	n.s.	n.s.	+	+	(Wong et al., 1995)
TDP-43^{Q331K}	1-1.5	3	> 24	+	+	+	+	+	+	(Arnold et al., 2013; Mitchell et al., 2015)

Prp-TDP43^{A315T}	n.a.	3-4	4.5-5.7	+	+	+	+	+	+	(Wegorzewska et al., 2009)
FUS^{P525L}	4	1	12	+	n.s.	n.s.	n.s.	+	n.s.	(Sharma et al., 2016)
FUS$^{dNLS/+}$	n.a.	10	>22	+	-	n.s.	n.s.	-	-	(Scekic-Zahirovic et al., 2016; Scekic-Zahirovic et al., 2017)
Alsin^{KO}	n.a.	12	>13	-	-	-	+	-	+	(Deng et al., 2007; Gautam et al., 2016)
UCHL1 null	n.a.	2.2	>6.6	+	-	+	+	-	+	(Jara et al., 2015) (Genç et al., 2016)
hPFN1^{G118V}	n.a.	4-4.5	5.7-7.7	+	+	+	+	+	+	(Fil et al., 2017)
Prp-PFN1^{C71G}	n.a.	4-5	6-8	+	+	-	-	+	+	(Yang et al., 2016)

References

Acevedo-Arozena, A., Kalmar, B., Essa, S., Ricketts, T., Joyce, P., Kent, R., et al. (2011). A comprehensive assessment of the SOD1G93A low-copy transgenic mouse, which models human amyotrophic lateral sclerosis. *Disease models & mechanisms* 4(5)**,** 686–700. doi: 10.1242/dmm.007237.

Arnold, E.S., Ling, S.-C., Huelga, S.C., Lagier-Tourenne, C., Polymenidou, M., Ditsworth, D., et al. (2013). ALS-linked TDP-43 mutations produce aberrant RNA splicing and adult-onset motor neuron disease without aggregation or loss of nuclear TDP-43. *Proceedings of the National Academy of Sciences* 110(8)**,** E736. doi: 10.1073/pnas.1222809110.

Bruijn, L.I., Becher, M.W., Lee, M.K., Anderson, K.L., Jenkins, N.A., Copeland, N.G., et al. (1997). ALS-linked SOD1 mutant G85R mediates damage to astrocytes and promotes rapidly progressive disease with SOD1-containing inclusions. *Neuron* 18(2)**,** 327-338. doi: 10.1016/s0896-6273(00)80272-x.

Chiu, A.Y., Zhai, P., Dal Canto, M.C., Peters, T.M., Kwon, Y.W., Prattis, S.M., et al. (1995). Age-Dependent Penetrance of Disease in a Transgenic Mouse Model of Familial Amyotrophic Lateral Sclerosis. *Molecular and Cellular Neuroscience* 6(4)**,** 349-362. doi: https://doi.org/10.1006/mcne.1995.1027.

Deng, H.-X., Zhai, H., Fu, R., Shi, Y., Gorrie, G.H., Yang, Y., et al. (2007). Distal axonopathy in an alsin-deficient mouse model. *Human molecular genetics* 16(23)**,** 2911-2920.

Fil, D., DeLoach, A., Yadav, S., Alkam, D., MacNicol, M., Singh, A., et al. (2017). Mutant Profilin1 transgenic mice recapitulate cardinal features of motor neuron disease. *Hum Mol Genet* 26(4)**,** 686-701. doi: 10.1093/hmg/ddw429.

Gautam, M., Jara, J.H., Sekerkova, G., Yasvoina, M.V., Martina, M., and Özdinler, P.H. (2016). Absence of alsin function leads to corticospinal motor neuron vulnerability via novel disease mechanisms. *Human molecular genetics* 25(6)**,** 1074-1087.

Genç, B., Jara, J.H., Schultz, M.C., Manuel, M., Stanford, M.J., Gautam, M., et al. (2016). Absence of UCHL 1 function leads to selective motor neuropathy. *Annals of clinical and translational neurology* 3(5)**,** 331-345. doi: 10.1002/acn3.298.

Gurney, M.E., Pu, H., Chiu, A.Y., Dal Canto, M.C., Polchow, C.Y., Alexander, D.D., et al. (1994). Motor neuron degeneration in mice that express a human Cu,Zn superoxide dismutase mutation. *Science (New York, N.Y.)* 264(5166)**,** 1772–1775. doi: 10.1126/science.8209258.

Jara, J.H., Genç, B., Cox, G.A., Bohn, M.C., Roos, R.P., Macklis, J.D., et al. (2015). Corticospinal motor neurons are susceptible to increased ER stress and display profound degeneration in the absence of UCHL1 function. *Cerebral cortex* 25(11)**,** 4259-4272.

Mitchell, J.C., Constable, R., So, E., Vance, C., Scotter, E., Glover, L., et al. (2015). Wild type human TDP-43 potentiates ALS-linked mutant TDP-43 driven progressive motor and cortical neuron degeneration with pathological features of ALS. *Acta Neuropathol Commun* 3**,** 36. doi: 10.1186/s40478-015-0212-4.

Özdinler, P.H., Benn, S., Yamamoto, T.H., Güzel, M., Brown, R.H., and Macklis, J.D. (2011). Corticospinal motor neurons and related subcerebral projection neurons undergo early and specific neurodegeneration in hSOD1G^{93}A transgenic ALS mice. *The Journal of neuroscience : the official journal of the Society for Neuroscience* 31(11)**,** 4166–4177. doi: 10.1523/jneurosci.4184-10.2011.

Ripps, M.E., Huntley, G.W., Hof, P.R., Morrison, J.H., and Gordon, J.W. (1995). Transgenic mice expressing an altered murine superoxide dismutase gene provide an animal model of amyotrophic lateral sclerosis. *Proc Natl Acad Sci U S A* 92(3)**,** 689-693. doi: 10.1073/pnas.92.3.689.

Scekic-Zahirovic, J., Oussini, H.E., Mersmann, S., Drenner, K., Wagner, M., Sun, Y., et al. (2017). Motor neuron intrinsic and extrinsic mechanisms contribute to the pathogenesis of FUS-associated amyotrophic lateral sclerosis. *Acta neuropathologica* 133(6)**,** 887-906. doi: 10.1007/s00401-017-1687-9.

Scekic-Zahirovic, J., Sendscheid, O., El Oussini, H., Jambeau, M., Sun, Y., Mersmann, S., et al. (2016). Toxic gain of function from mutant FUS protein is crucial to trigger cell autonomous motor neuron loss. *The EMBO journal* 35(10)**,** 1077-1097. doi: 10.15252/embj.201592559.

Sharma, A., Lyashchenko, A.K., Lu, L., Nasrabady, S.E., Elmaleh, M., Mendelsohn, M., et al. (2016). ALS-associated mutant FUS induces selective motor neuron degeneration through toxic gain of function. *Nature communications* 7**,** 10465-10465. doi: 10.1038/ncomms10465.

Wegorzewska, I., Bell, S., Cairns, N.J., Miller, T.M., and Baloh, R.H. (2009). TDP-43 mutant transgenic mice develop features of ALS and frontotemporal lobar degeneration. *Proceedings of the National Academy of Sciences* 106(44)**,** 18809. doi: 10.1073/pnas.0908767106.

Wong, P.C., Pardo, C.A., Borchelt, D.R., Lee, M.K., Copeland, N.G., Jenkins, N.A., et al. (1995). An adverse property of a familial ALS-linked SOD1 mutation causes motor neuron disease characterized by vacuolar degeneration of mitochondria. *Neuron* 14(6)**,** 1105-1116. doi: 10.1016/0896-6273(95)90259-7.

Yang, C., Danielson, E.W., Qiao, T., Metterville, J., Brown, R.H., Landers, J.E., et al. (2016). Mutant PFN1 causes ALS phenotypes and progressive motor neuron degeneration in mice by a gain of toxicity. *Proceedings of the National Academy of Sciences* 113(41)**,** E6209. doi: 10.1073/pnas.1605964113.
