# Supplementary material for: Exciting Complexity: The Role of Motor Circuit Elements in ALS Pathophysiology
Source: Front Neurosci. 2020 Jun 17;14:573. doi: 10.3389/fnins.2020.00573 (PMC7311855; doi:10.3389/fnins.2020.00573)
Supplement: Supplementary file 2 [file Table_2.pdf]

Supplementary Table 2. Summary of excitability changes of peripheral nerves in ALS patients. Abbreviations: asymp., asymptomatic; CMAP, compound muscle action potential; EMG, electromyography; fALS, familial ALS; FPs, fasciculation potentials; H'H ratio, H' response to the maximum H-reflex response; MUs, motor units; n.s., not specified; MUAP, muscle unit action potentials; NCS, nerve conduction studies; SOD1, superoxide dismutase 1; sALS, sporadic ALS; TEd, depolarizing threshold electrotonus; TEh, hyperpolarizing threshold electrotonus; τ_{SD}, strength-duration constant.

region	ALS type	age (average)	method	finding	references
**Peripheral nerves**	sALS	51 years	NCS in median, sural and peroneal nerves; EMG of interosseus, biceps brachii, vastus lateralis and tibialis anterior muscles	hyperexcitability: FP ↑, double discharging MUs ↑	(Kostera-Pruszczyk et al., 2002)
	sALS	63 years	NCS of median nerve and CMAP recordings of abductor pollicis brevis<br><br>threshold tracking and current-threshold measurements of median nerve	CMAP ↓<br>axonal hyperexcitability: τ_{SD} ↑ (CMAP > 5 mV group), TEd ↑, supernormality ↑	(Kanai et al., 2006)
		59.6 years		CMAP ↓<br>axonal hyperexcitability: τ_{SD} ↑, TEd ↑, TEh ↑<br>superexcitability ↑	(Vucic and Kiernan, 2006)
	fALS	58 years		CMAP ↓<br>axonal hyperexcitability: τ_{SD} ↑	(Vucic and Kiernan, 2009)
	sALS	59 years			
	fALS (asymp. SOD1)	40 years		axonal excitability ↔, τ_{SD} ↔	
	n.s.	59.6 years	threshold tracking measurements of ulnar nerve	axonal hyperexcitability (7 patients): TEd ↑<br>axonal hypoexcitability (4 patients): TEd ↓	(Bostock et al., 1995)
		n.s.	threshold tracking measurements of median nerve	axonal hyperexcitability: TEd ↑, TEh ↑	(Horn et al., 1996)
		59.1 years	NCS of median nerve and CMAP recordings of abductor pollicis brevis; threshold tracking of median nerve	CMAP ↓<br>axonal hyperexcitability: τ_{SD} ↑, rheobase ↓	(Mogyoros et al., 1998)
		64 years	NCS of median nerve and CMAP recordings of abductor pollicis brevis	CMAP ↓, conduction velocity ↓	(Tamura et al., 2006)
			latent addition with threshold tracking of the median nerve	axonal hyperexcitability: nodal persistent Na^{+} conductance ↑, TEd ↑	
		66 years	NCS of median nerve and CMAP recordings of abductor pollicis brevis; threshold tracking of median nerve at wrist (nerve trunk) and at abductor pollicis brevis (distal axons)	CMAP ↓<br>axonal hyperexcitability: TEd ↑, TEd ↑ (distal axons) > TEd ↑ (nerve trunk)	(Nakata et al., 2006)
		54.7 years	single MUAP recordings of the brachial biceps	hyperexcitability: double discharge firing MUs ↑	(Piotrkiewicz et al., 2008)
		64.0 years	EMG of tibialis anterior	hyperexcitability: complex FPs ↑	(de Carvalho and Swash, 2013)
		46.3 years	soleus H-reflex measurement with EMG	recurrent inhibition ↓, H'/H ratio ↓	(Raynor and Shefner, 1994)
	Riluzole treated ALS patients	54 years	threshold tracking and single MUAP recordings of the abductor pollicis brevis and abductor digiti minimi	CMAP ↓<br>axonal hypoexcitability: rheobase ↑, threshold for single MU ↑, axonal hyperexcitability: TEd ↑, superexcitability ↑	(Howells et al., 2018)

References

Bostock, H., Sharief, M.K., Reid, G., and Murray, N.M. (1995). Axonal ion channel dysfunction in amyotrophic lateral sclerosis. *Brain* 118 (Pt 1), 217-225. doi: 10.1093/brain/118.1.217.

de Carvalho, M., and Swash, M. (2013). Fasciculation potentials and earliest changes in motor unit physiology in ALS. *J Neurol Neurosurg Psychiatry* 84(9), 963-968. doi: 10.1136/jnnp-2012-304545.

Horn, S., Quasthoff, S., Grafe, P., Bostock, H., Renner, R., and Schrank, B. (1996). Abnormal axonal inward rectification in diabetic neuropathy. *Muscle Nerve* 19(10), 1268-1275. doi: 10.1002/mus.880191002.

Howells, J., Matamala, J.M., Park, S.B., Garg, N., Vucic, S., Bostock, H., et al. (2018). In vivo evidence for reduced ion channel expression in motor axons of patients with amyotrophic lateral sclerosis. *The Journal of physiology* 596(22), 5379-5396. doi: 10.1113/JP276624.

Kanai, K., Kuwabara, S., Misawa, S., Tamura, N., Ogawara, K., Nakata, M., et al. (2006). Altered axonal excitability properties in amyotrophic lateral sclerosis: impaired potassium channel function related to disease stage. *Brain* 129(4), 953-962. doi: 10.1093/brain/awl024.

Kostera-Pruszczyk, A., Niebroj-Dobosz, I., Emeryk-Szajewska, B., Karwanska, A., and Rowinska-Marcinska, K. (2002). Motor unit hyperexcitability in amyotrophic lateral sclerosis vs amino acids acting as neurotransmitters. *Acta Neurol Scand* 106(1), 34-38. doi: 10.1034/j.1600-0404.2002.00149.x.

Mogyoros, I., Kiernan, M.C., Burke, D., and Bostock, H. (1998). Strength-duration properties of sensory and motor axons in amyotrophic lateral sclerosis. *Brain* 121 (Pt 5), 851-859. doi: 10.1093/brain/121.5.851.

Nakata, M., Kuwabara, S., Kanai, K., Misawa, S., Tamura, N., Sawai, S., et al. (2006). Distal excitability changes in motor axons in amyotrophic lateral sclerosis. *Clinical Neurophysiology* 117(7), 1444-1448. doi: https://doi.org/10.1016/j.clinph.2006.04.005.

Piotrkiewicz, M., Kudina, L., Mierzejewska, J., and Hausmanowa-Petrusewicz, I. (2008). Analysis of double discharges in amyotrophic lateral sclerosis. *Muscle Nerve* 38(1), 845-854. doi: 10.1002/mus.20997.

Raynor, E.M., and Shefner, J.M. (1994). Recurrent inhibition is decreased in patients with amyotrophic lateral sclerosis. *Neurology* 44(11), 2148-2153. doi: 10.1212/wnl.44.11.2148.

Tamura, N., Kuwabara, S., Misawa, S., Kanai, K., Nakata, M., Sawai, S., et al. (2006). Increased nodal persistent Na+ currents in human neuropathy and motor neuron disease estimated by latent addition. *Clinical Neurophysiology* 117(11), 2451-2458. doi: https://doi.org/10.1016/j.clinph.2006.07.309.

Vucic, S., and Kiernan, M.C. (2006). Axonal excitability properties in amyotrophic lateral sclerosis. *Clinical Neurophysiology* 117(7), 1458-1466. doi: https://doi.org/10.1016/j.clinph.2006.04.016.

Vucic, S., and Kiernan, M.C. (2009). Upregulation of persistent sodium conductances in familial ALS. *Journal of Neurology, Neurosurgery & Psychiatry* 81(2), 222-227. doi: 10.1136/jnnp.2009.183079.
