# Supplementary material for: Exciting Complexity: The Role of Motor Circuit Elements in ALS Pathophysiology
Source: Front Neurosci. 2020 Jun 17;14:573. doi: 10.3389/fnins.2020.00573 (PMC7311855; doi:10.3389/fnins.2020.00573)
Supplement: Supplementary file 3 [file Table_3.pdf]

Supplementary Table 3. Altered molecular composition of astrocytes in ALS. Abbreviations: 2D-HPLC, two-dimensional high performance liquid chromatography; ACM, astrocyte conditioned medium; ALS, amyotrophic lateral sclerosis; Cx43, connexin 43; EAAT2, excitatory amino acid transporter 2; ELISA, enzyme-linked immunosorbent assay; ES, embryonic stem; ESC, embryonic stem cell; ER, endoplasmic reticulum; fALS, familial ALS; GDNF, glial-derived neurotrophic factor; IFN, interferon; IFN- αR1, interferon α receptor 1; IHC, immunohistochemistry; IPSCs, induced pluripotent stem cells; IF, immunofluorescence; iNOS, inducible nitric oxide synthase; ISG, IFN-stimulated gene; Kir 4.1, inward-rectifying potassium channel 4.1; LC-HRMS, liquid chromatography coupled with high resolution mass spectrometry; LMN, lower motor neuron; LDH, lactate dehydrogenase; mPTP-I, mitochondria permeability transition pore inhibitor; NMDA, N-methyl-D-aspartate; n.a, not applicable; n.s., not specified; NOS, nitric oxide synthase; NOX2, NADPH oxidase 2; PDC, parkinsonism dementia complex; PDG2, Prostaglandin D2; RCR, respiratory control ratio; ROS, reactive oxygen species; RT RT-qPCR, Real-Time quantitative reverse transcription polymerase chain reaction; SOD1, superoxide dismutase 1; sALS, sporadic ALS; SOCE, ER store-operated calcium entry; TGF-β, transforming growth factor β; TNF, neurotrophic factor; TNFR1, TNF receptor 1; WB, western blotting.

	**ALS model**	**disease stage**	**region**	**method of investigation**	**findings**	**references**
**rodent models of ALS**	ALS-PDC	symptomatic	motor cortex spinal cord	WB and IHC	GLT-1 ↓	(Wilson et al., 2003)
	$SOD1^{G85R}$	end-stage	spinal cord	WB		(Bruijn et al., 1997)
	$SOD1^{G93A}$	presymptomatic, symptomatic and end-stage	spinal cord ventral horn	IHC		(Howland et al., 2002)
	TDP-43	disease onset and symptomatic	spinal cord	IHC		(Tong et al., 2013)
	$SOD1^{G93A}$	presymptomatic, symptomatic and end-stage	spinal cord ventral horn	IF and WB	Kir 4.1 ↓	(Kaiser et al., 2006)
		symptomatic	brainstem and cortex	IF, WB and whole-cell patch-clamp recordings in cultured cortical astrocytes		(Bataveljic et al., 2012)
	$SOD1^{G93A}$	presymptomatic, symptomatic and end-stage	spinal cord ventral horn	WB	AQP4 ↑	(Kaiser et al., 2006)
		symptomatic	brainstem and cortex	WB and IHC		(Bataveljic et al., 2012)
	$SOD1^{G93A}$	presymptomatic	lumbar spinal cord	transcriptomics, lactate level in spinal cord and astrocyte-conditioned medium and qPCR of LMN	lactate release ↓	(Ferraiuolo et al., 2011)
		neonatal (P2-3)	spinal cord	LC-HRMS metabolomics of astrocyte mono-cultures and astrocyte-LMN (WT) co-cultures upon glutamate treatment		(Madji Hounoum et al., 2017)
	$SOD1^{G93A}$	end-stage	motor cortex and spinal cord	IF,WB, Fura-2 indicated Ca^{2+} imaging combined with GAP26 treatment	Cx43 ↑	(Almad et al., 2016)
	$SOD1^{G93A}$	neonatal (P1-2)	brain and spinal cord	IHC astrocyte-MN co-culture, RCR indicating oxygen consumption measured before and after NOS inhibitor	NOS ↑	(Cassina et al., 2008)
	$SOD1^{G93A}$	presymptomatic and symptomatic	lumbar spinal cord	2D-HPLC	D-Serine ↑	(Sasabe et al., 2012)
		presymptomatic, symptomatic and end-stage	lumbar spinal cord	IF, D-Serine level measured by chemiluminescence, viability assay of LMN/UMN culture upon treatment of D-Serine or antagonist to the glycine-binding site of NMDARs		(Sasabe et al., 2007)

	SOD1^{G93A}	disease onset and presymptomatic	spinal cord	WB, Fura-4 indicated Ca^{2+} imaging and luciferase assay	ER Ca^{2+} release↑<br>ER Ca^{2+} storage ↑<br>ATP secretion ↑<br>Ca^{2+} influx dysregulation	(Kawamata et al., 2014)
		neonatal (P2)	cortex			
	SOD1^{G93A}	symptomatic	motor cortex	*in vivo* awakening two-photo Ca^{2+} imaging and Ca^{2+} imagining with mPTP-I treatment to acute cortical slices	Ca^{2+} activity ↑	(Agarwal et al., 2017)
	SOD1^{G93A}	symptomatic and end-stage	spinal cord	IF and WB	TGF-β1 ↑	(Endo et al., 2015)
	SOD1^{G85R} and SOD1^{G37R}	end-stage				
	SOD1^{G93A}	neonatal (P1-3)	n.s.	IF of human ESC-derived MNs co-cultured with astrocyte and viability assay of MNs with PDG2 receptor antagonist MK0524 treatment	PDG2 ↑	(Di Giorgio et al., 2008)
	SOD1^{G93A}	disease onset and symptomatic	spinal cord	WB, ELISA and IF	IFN- γ ↑	(Aebischer et al., 2011)
		neonatal (P1-2)		WB, ELISA, viability assay of LMN (wt)-astrocyte co-culture and IFN-γ antagonist treatment		
	SOD1^{G93A}	presymptomatic	lumbar spinal cord	RNA-seq, qPCR, ESC-derived (E15) MNs treated with the potent ER stress inducer	IFN-α ↑<br>ISG ↑	(Wang et al., 2011)
	SOD1^{G93A}	disease onset and symptomatic	spinal cord	RT RT-qRCR of GDNF, TNF and TNFR1, reduction and increase of TNFR1 expression, IHC	GDNF ↑<br>TNF ↑<br>TNFR1 ↑	(Brambilla et al., 2016)
**human-derived astrocytes**	fALS (*SOD1*^{D90A} mutation)	n.a.	IPSC-derived astrocytes	qPCR and WB	Kir 4.1 ↓	(Kelley et al., 2018)
	fALS (*SOD1 or C9orf72*) and sALS	n.a.	IPSC-derived astrocytes	IF, WB, Fura-2 indicated Ca^{2+} imaging rescued with Cx43 blocker GAP26 treatment	Cx43 ↑	(Almad et al., 2016)
	SOD1^{G37R}	fetal	cerebral cortex obtained from fetal human brain culture	WB, IHC, NOX2 inhibitor or antioxidant treatment	ROS↑<br>NOX2↑<br>iNOS ↑	(Marchetto et al., 2008)
**ALS patients**	fALS and sALS	post-mortem (mean: 61 yrs)	motor cortex and lumbar spinal cord	WB	EAAT2 ↓	(Rothstein et al., 1995)
	fALS (*SOD1*^{A4V} mutation) and sALS	post-mortem	lumbar spinal cord	IHC	D-Serine ↑	(Sasabe et al., 2007)
	fALS and sALS	post-mortem	lumbar spinal cord	WB and IF	IFN-α ↑<br>ISG ↑	(Wang et al., 2011)
	sALS	post-mortem	spinal cord	RT-qPCR	GDNF ↑<br>TNF ↑<br>TNFR1 ↑	(Brambilla et al., 2016)

References

Aebischer, J., Cassina, P., Otsmane, B., Moumen, A., Seilhean, D., Meininger, V., et al. (2011). IFNγ triggers a LIGHT-dependent selective death of motoneurons contributing to the non-cell-autonomous effects of mutant SOD1. *Cell death and differentiation* 18(5)**,** 754-768. doi: 10.1038/cdd.2010.143.

Agarwal, A., Wu, P.H., Hughes, E.G., Fukaya, M., Tischfield, M.A., Langseth, A.J., et al. (2017). Transient Opening of the Mitochondrial Permeability Transition Pore Induces Microdomain Calcium Transients in Astrocyte Processes. *Neuron* 93(3)**,** 587-605.e587. doi: 10.1016/j.neuron.2016.12.034.

Almad, A.A., Doreswamy, A., Gross, S.K., Richard, J.-P., Huo, Y., Haughey, N., et al. (2016). Connexin 43 in astrocytes contributes to motor neuron toxicity in amyotrophic lateral sclerosis. *Glia* 64(7)**,** 1154-1169. doi: 10.1002/glia.22989.

Bataveljic, D., Nikolic, L., Milosevic, M., Todorovic, N., and Andjus, P.R. (2012). Changes in the astrocytic aquaporin-4 and inwardly rectifying potassium channel expression in the brain of the amyotrophic lateral sclerosis SOD1(G93A) rat model. *Glia* 60(12)**,** 1991-2003. doi: 10.1002/glia.22414.

Brambilla, L., Guidotti, G., Martorana, F., Iyer, A.M., Aronica, E., Valori, C.F., et al. (2016). Disruption of the astrocytic TNFR1-GDNF axis accelerates motor neuron degeneration and disease progression in amyotrophic lateral sclerosis. *Hum Mol Genet* 25(14)**,** 3080-3095. doi: 10.1093/hmg/ddw161.

Bruijn, L.I., Becher, M.W., Lee, M.K., Anderson, K.L., Jenkins, N.A., Copeland, N.G., et al. (1997). ALS-linked SOD1 mutant G85R mediates damage to astrocytes and promotes rapidly progressive disease with SOD1-containing inclusions. *Neuron* 18(2)**,** 327-338. doi: 10.1016/s0896-6273(00)80272-x.

Cassina, P., Cassina, A., Pehar, M., Castellanos, R., Gandelman, M., de Leon, A., et al. (2008). Mitochondrial dysfunction in SOD1G93A-bearing astrocytes promotes motor neuron degeneration: prevention by mitochondrial-targeted antioxidants. *J Neurosci* 28(16)**,** 4115-4122. doi: 10.1523/jneurosci.5308-07.2008.

Di Giorgio, F.P., Boulting, G.L., Bobrowicz, S., and Eggan, K.C. (2008). Human embryonic stem cell-derived motor neurons are sensitive to the toxic effect of glial cells carrying an ALS-causing mutation. *Cell Stem Cell* 3(6)**,** 637-648. doi: 10.1016/j.stem.2008.09.017.

Endo, F., Komine, O., Fujimori-Tonou, N., Katsuno, M., Jin, S., Watanabe, S., et al. (2015). Astrocyte-derived TGF-beta1 accelerates disease progression in ALS mice by interfering with the neuroprotective functions of microglia and T cells. *Cell Rep* 11(4)**,** 592-604. doi: 10.1016/j.celrep.2015.03.053.

Ferraiuolo, L., Higginbottom, A., Heath, P.R., Barber, S., Greenald, D., Kirby, J., et al. (2011). Dysregulation of astrocyte-motoneuron cross-talk in mutant superoxide dismutase 1-related amyotrophic lateral sclerosis. *Brain : a journal of neurology* 134(Pt 9)**,** 2627-2641. doi: 10.1093/brain/awr193.

Howland, D.S., Liu, J., She, Y., Goad, B., Maragakis, N.J., Kim, B., et al. (2002). Focal loss of the glutamate transporter EAAT2 in a transgenic rat model of SOD1 mutant-mediated amyotrophic lateral sclerosis (ALS). *Proc Natl Acad Sci U S A* 99(3)**,** 1604-1609. doi: 10.1073/pnas.032539299.

Kaiser, M., Maletzki, I., Hulsmann, S., Holtmann, B., Schulz-Schaeffer, W., Kirchhoff, F., et al. (2006). Progressive loss of a glial potassium channel (KCNJ10) in the spinal cord of the SOD1 (G93A) transgenic mouse model of amyotrophic lateral sclerosis. *J Neurochem* 99(3)**,** 900-912. doi: 10.1111/j.1471-4159.2006.04131.x.

Kawamata, H., Ng, S.K., Diaz, N., Burstein, S., Morel, L., Osgood, A., et al. (2014). Abnormal intracellular calcium signaling and SNARE-dependent exocytosis contributes to SOD1G93A astrocyte-mediated toxicity in amyotrophic lateral sclerosis. *J Neurosci* 34(6)**,** 2331-2348. doi: 10.1523/jneurosci.2689-13.2014.

Kelley, K.W., Ben Haim, L., Schirmer, L., Tyzack, G.E., Tolman, M., Miller, J.G., et al. (2018). Kir4.1-Dependent Astrocyte-Fast Motor Neuron Interactions Are Required for Peak Strength. *Neuron* 98(2)**,** 306-319.e307. doi: 10.1016/j.neuron.2018.03.010.

Madji Hounoum, B., Mavel, S., Coque, E., Patin, F., Vourc'h, P., Marouillat, S., et al. (2017). Wildtype motoneurons, ALS-Linked SOD1 mutation and glutamate profoundly modify astrocyte metabolism and lactate shuttling. *Glia* 65(4)**,** 592-605. doi: 10.1002/glia.23114.

Marchetto, M.C.N., Muotri, A.R., Mu, Y., Smith, A.M., Cezar, G.G., and Gage, F.H. (2008). Non-Cell-Autonomous Effect of Human SOD1G37R Astrocytes on Motor Neurons Derived from Human Embryonic Stem Cells. *Cell Stem Cell* 3(6)**,** 649-657. doi: https://doi.org/10.1016/j.stem.2008.10.001.

Rothstein, J.D., Van Kammen, M., Levey, A.I., Martin, L.J., and Kuncl, R.W. (1995). Selective loss of glial glutamate transporter GLT-1 in amyotrophic lateral sclerosis. *Ann Neurol* 38(1)**,** 73-84. doi: 10.1002/ana.410380114.

Sasabe, J., Chiba, T., Yamada, M., Okamoto, K., Nishimoto, I., Matsuoka, M., et al. (2007). D-serine is a key determinant of glutamate toxicity in amyotrophic lateral sclerosis. *Embo j* 26(18)**,** 4149-4159. doi: 10.1038/sj.emboj.7601840.

Sasabe, J., Miyoshi, Y., Suzuki, M., Mita, M., Konno, R., Matsuoka, M., et al. (2012). D-amino acid oxidase controls motoneuron degeneration through D-serine. *Proc Natl Acad Sci U S A* 109(2)**,** 627-632. doi: 10.1073/pnas.1114639109.

Tong, J., Huang, C., Bi, F., Wu, Q., Huang, B., Liu, X., et al. (2013). Expression of ALS-linked TDP-43 mutant in astrocytes causes non-cell-autonomous motor neuron death in rats. *Embo j* 32(13)**,** 1917-1926. doi: 10.1038/emboj.2013.122.

Wang, R., Yang, B., and Zhang, D. (2011). Activation of interferon signaling pathways in spinal cord astrocytes from an ALS mouse model. *Glia* 59(6)**,** 946-958. doi: 10.1002/glia.21167.

Wilson, J.M.B., Khabazian, I., Pow, D.V., Craig, U.K., and Shaw, C.A. (2003). Decrease in glial glutamate transporter variants and excitatory amino acid receptor down-regulation in a murine model of ALS-PDC. *NeuroMolecular Medicine* 3(2)**,** 105-117. doi: 10.1385/nmm:3:2:105.
